# Mate pair sequencing outperforms fluorescence in situ hybridization in the genomic characterization of multiple myeloma

**DOI:** 10.1038/s41408-019-0255-z

**Published:** 2019-12-16

**Authors:** James Smadbeck, Jess F. Peterson, Kathryn E. Pearce, Beth A. Pitel, Andrea Lebron Figueroa, Michael Timm, Dragan Jevremovic, Min Shi, A. Keith Stewart, Esteban Braggio, Daniel L. Riggs, P. Leif Bergsagel, George Vasmatzis, Hutton M. Kearney, Nicole L. Hoppman, Rhett P. Ketterling, Shaji Kumar, S. Vincent Rajkumar, Patricia T. Greipp, Linda B. Baughn

**Affiliations:** 1 Center for Individualized Medicine-Biomarker Discovery, Mayo Clinic, Rochester, MN USA; 2 Division of Laboratory Genetics, Department of Laboratory Medicine and Pathology, Mayo Clinic, Rochester, MN USA; 3 Division of Hematopathology, Department of Laboratory Medicine and Pathology, Mayo Clinic, Rochester, MN USA; 4 Division of Hematology, Department of Internal Medicine, Mayo Clinic, Scottsdale, AZ USA; 5 Division of Hematology, Department of Internal Medicine, Mayo Clinic, Rochester, MN USA

**Keywords:** Cancer genomics, Cancer genetics

## Abstract

Fluorescence in situ hybridization (FISH) is currently the gold-standard assay to detect recurrent genomic abnormalities of prognostic significance in multiple myeloma (MM). Since most translocations in MM involve a position effect with heterogeneous breakpoints, we hypothesize that FISH has the potential to miss translocations involving these regions. We evaluated 70 bone marrow samples from patients with plasma cell dyscrasia by FISH and whole-genome mate-pair sequencing (MPseq). Thirty cases (42.9%) displayed at least one instance of discordance between FISH and MPseq for each primary and secondary abnormality evaluated. Nine cases had abnormalities detected by FISH that went undetected by MPseq including 6 tetraploid clones and three cases with missed copy number abnormalities. In contrast, 19 cases had abnormalities detected by MPseq that went undetected by FISH. Seventeen were *MYC* rearrangements and two were 17p deletions. MPseq identified 36 *MYC* abnormalities and 17 (50.0% of *MYC* abnormal group with FISH results) displayed a false negative FISH result. MPseq identified 10 cases (14.3%) with IgL rearrangements, a recent marker of poor outcome, and 10% with abnormalities in genes associated with lenalidomide response or resistance. In summary, MPseq was superior in the characterization of rearrangement complexity and identification of secondary abnormalities demonstrating increased clinical value compared to FISH.

## Introduction

Multiple myeloma (MM) is a plasma cell neoplasm (PCN) representing the second most common hematopoietic malignancy and accounts for ~20% of all hematologic cancer related deaths in the United States^1^. During the last decade there have been remarkable improvements in the treatment of patients with MM that have resulted in increased survival, including immunomodulatory compounds, proteasome inhibitors, and immunotherapeutic approaches such as monoclonal antibodies^2^. Paralleling the advances in novel therapeutic strategies, characterization of the genomic complexities of MM have significantly improved with the implementation of next-generation sequencing (NGS), thus enabling the identification of novel single nucleotide variants (SNV), structural rearrangements and copy number abnormalities (CNA)^3–11^. Comprehensive genomic characterization studies such as the Multiple Myeloma Research Foundation (MMRF) CoMMpass Trial and other research studies are necessary for the discovery of novel variants of clinical significance that may lead to improved treatment approaches and prognostication strategies^12,13^.

In contrast to the use of genome-wide NGS strategies employed in the research/investigational trial setting, most clinical genomics laboratories rely upon traditional cytogenetic methodologies such as conventional chromosome studies and fluorescence in situ hybridization (FISH) to characterize recurrent cytogenetic abnormalities of prognostic significance. High-risk cytogenetic abnormalities as defined by the Mayo Clinic mSMART 3.0 algorithm^14^ include t(4;14), t(14;16), t(14;20) translocations, 17p deletions and 1q gains, while standard-risk cytogenetic abnormalities include hyperdiploidy (gains of odd-numbered chromosomes), t(11;14) and t(6;14) translocations^15,16^. A limited number of laboratories evaluate for *MYC* and t(6;14) rearrangements, and detection of *IGK* and *IgL* rearrangements is not routinely performed in the clinical setting^15^. Although FISH assays have high sensitivity, are relatively inexpensive compared to NGS techniques and provide input for risk stratification^17^, several limitations exist. They allow for the interrogation of only the regions for which FISH probes are available and multiple FISH probes are needed in order to be comprehensive, with each probe requiring a resource-consuming validation. More importantly, FISH has the potential to miss cryptic abnormalities, including rearrangements that result in a position effect due to juxtaposition of enhancers near oncogenes^18–22^. Since many translocations identified in MM involve a position effect (i.e., IGH and *MYC*) with heterogeneous breakpoints^10,23,24^ and some CNAs may be cryptic, we hypothesize that some clinical FISH probes used in the characterization of PCNs have a high rate of false negative FISH results.

To test this hypothesis, we evaluated the performance of a genome wide mate-pair sequencing (MPseq) assay in comparison to FISH panel testing for MM. Since MPseq utilizes long input DNA (2–5 Kb) followed by circularization and fragmentation to the size of paired-end fragments (200–500 bp) that are sequenced at reduced depth, this assay is designed to detect structural rearrangements and CNAs throughout the genome resulting in a cost-effective strategy amenable to a clinical genomics laboratory. Furthermore, as MPseq has higher resolution than FISH and is not limited to specific genomic footprints for interrogation, this assay could provide an alternative technique to comprehensively detect structural rearrangements and CNAs in a single assay. Herein we describe the performance, along with the added clinical utility, of MPseq in 70 samples previously characterized by FISH to detect chromosome rearrangements and CNAs in patients with a PCN.

## Methods

### Patient samples

All samples were referred to the Mayo Clinic Genomics Laboratory as part of routine clinical testing and further evaluated by MPseq as part of a Mayo Clinic Institutional Review Board approved study. There were multiple sources of samples obtained either from fresh or frozen whole bone marrow (BM), or from fixed cell pellets (FCP) from an abnormal BM chromosome study. Some specimens had undergone plasma cell enrichment from fresh whole BM that was either flow sorted or subjected to CD138+ magnetic-enrichment from patients that had an abnormal plasma cell FISH result. Additional methodology, including conventional chromosome analysis and flow cytometry are included in supplemental data.

### Fluorescence in situ hybridization

Plasma cell proliferative disorder FISH (PCPDF) of immunoglobulin (cIg)-stained positive PCs studies were performed as previously described^25^ using the following probes to detect primary and secondary MM abnormalities: monosomy 13 or 13q deletion (Abbott Molecular, Abbott Park, IL), monosomy 17 or *TP53* deletion (Abbott Molecular), trisomy 3, 7, 9 or 15 (Abbott Molecular), 1q gain (in house, custom developed), *MYC* rearrangement (Abbott Molecular), IGH rearrangement (in house, custom developed), t(11;14) *CCND1/IGH* (Abbott Molecular), t(4;14)(p16.3;q32) *FGFR3*/*IGH* (Abbott Molecular), t(6;14)(p21;q32) *CCND3*/*IGH* (Abbott Molecular), t(14;16)(q32;q23) *IGH*/*MAF* (Abbott Molecular), and t(14;20)(q32;q12) *IGH*/*MAFB* (Abbott Molecular). The PCN FISH panel is indicated in supplemental Table 1 with footprints and probe source shown in supplemental Table 2.

### Plasma cell enrichment

BM cells (20 × 10^6^) were lysed in ACK lysis buffer for 5 min. This was followed by 2 wash steps in PBS (lyse-wash procedure) and the cell pellet was re-suspended in 3% BSA/PBS. 10 × 10^6^ cells were then incubated for 15 min with the following antibodies: CD19-PerCP 5.5 (clone SJ25C1, BD Biosciences), CD38-APC (clone REA671, Miltenyi Biotec), CD45-BB515 (clone HI30, BD Biosciences), CD56-PE-Cy7 (clone NCAM16.2, BD Biosciences), CD138-BV421 (clone MI15, BD Biosciences), and CD319-PE (clone REA150, Miltenyi Biotec). The specimen was centrifuged and re-suspended in 1.5 mL of PBS. Sorting was performed on BD FACSMelody cell sorter (BD Biosciences, San Jose, CA). Sorting streams were defined for each case separately, using gates to include CD138-positive, CD319-positive, CD38-bright, CD56-positive and/or CD45-negative plasma cells, and separate them from normal plasma cells. A minimum of 2 × 10^5^ cells were collected, with the purity of at least 95%, verified by Kaluza software (Beckman Coulter Life Sciences, Indianapolis, IN). In some cases, plasma cells were separated by positive selection using CD138-coated magnetic beads (MACS; Miltenyi Biotec, CA) in a RoboSep system (STEMCELL Technology, Canada) as described in Jang et al.^26^.

### DNA extraction and library preparation

DNA extraction and mate pair library preparation methods have been previously described^18,27,28^. Briefly, DNA was isolated using either the Qiagen Puregene extraction kit (for samples < 2 mL), Autopure LS Automated high quality DNA extraction (for samples > 2 mL) or the QIAmp Tissue kit for fixed cell pellet samples. DNA was processed using the Illumina Nextera Mate Pair library preparation kit and sequenced on the Illumina HiSeq 2500 in rapid run mode as described in Aypar, et al.^18^. Pooled libraries were hybridized onto a flow cell (2 samples per lane) and sequenced using 101-basepair reads and paired end sequencing.

### Structural variant bioinformatics pipeline and visualization

The sequencing data was analyzed for the detection of structural variants (SVs), which are large genomic changes (>30Kb) that involve breakpoint junctions and/or CNAs. The sequencing data was mapped to the reference genome (GRCh38) using BIMA^29^ and the output was analyzed using SVAtools. This set of algorithms can detect and report the breakpoint locations of both junctions and CNAs at high resolution and accuracy (Schematic in supplemental Fig. 1)^18,27,28^. Junctions and CNVs were graphically illustrated using genome, junction and region plots as previously described^18,27,30^.

## Results

### Patient characteristics

A total of 70 cases referred to the Mayo Clinic Genomics Laboratory for routine clinical PCN FISH testing were selected for further evaluation by MPseq (Tables 1, 2 and supplemental Table 3). Criteria for inclusion included the type of primary cytogenetic abnormality to ensure representation of each recurring rearrangement and sample source to evaluate various methods of sample attainment, including PC enrichment (Tables 1, 2). The median age was 66 years (range 42–88) demonstrating male predominance with a 1.3:1 (M:F) ratio. Fifty-seven cases (81.4%) had either a diagnosis of MM (*N* = 35) or a reason for referral (RFR) of MM or PCN indicated at the time of clinical testing (*N* = 22) (Tables 1, 2). Of thirty-five cases with complete clinical data, 13 (37.1%) were newly diagnosed (ND) and 22 (62.9%) had relapsed and/or refractory disease (RR).

**Table 1:** Patient cohort.

	Site	Sex	Age (years)	Dx or RFR^a^	ND RR	% PC	Light chain	Sample type	Primary abnormality (FISH)	FISH
1	MCL	M	77	MM^a^	U	85	Kappa	FCP	11;14	nuc ish(MYC,RB1,LAMP1)x1,(CCND1-XT,IGH-XT)x3(CCND1-XT con IGH-XTx2)
2	MAYO	F	65	MM	ND	23	Lambda	Sort	11;14	nuc ish(TP73x2,1q22x3),(5′MYCx2,3’MYCx1)(5′MYC con 3′MYCx1),(CCND1-XT,IGH-XT)x3(CCND1-XT con IGH-XTx2)
3	MCL	F	70	PCN^a^	U	19	Kappa	Sort	11;14	nuc ish(MYCx2)(5′MYC sep 3′MYCx1),(CCND1-XT,IGH-XT)x3(CCND1-XT con IGH-XTx2)/(CCND1-XT,IGH-XT)x4(CCND1-XT con IGH-XTx3),(RB1,LAMP1)x1,(TP53x1,D17Z1x2)
4	MAYO	M	71	MM	ND	37	Lambda	Sort	11;14	nuc ish(TP73x2,1q22x3),(CCND1-XT,IGH-XT)x4–5(CCND1-XT con IGH-XTx3–4)/(CCND1-XTx1,CCND1-XT amp,IGH-XTx1,IGH-XT amp)(CCND1-XT amp con IGH-XT amp),(RB1,LAMP1)x1
5	MCL	M	69	MM^a^	U	50	Kappa	Sort	11;14	nuc ish(TP73x2,1q22x5),(D3Z1,D9Z1,D15Z4)x3,(CCND1-XT,IGH-XT)x3(CCND1-XT con IGH-XTx2),(RB1,LAMP1)x1
6	MAYO	M	63	MM	RR	28	Kappa	Sort	11;14	nuc ish(TP73x1,1q22x3–4)/(TP73x2,1q22x6),(D3Z1,D7Z1,D9Z1,D15Z4)x4,(5′MYCx3,3′MYCx2)(5′MYC con 3′MYCx2)/(5′MYCx6,3′MYCx4)(5′MYC con 3′MYCx4),(CCND1-XT,IGH-XT)x3(CCND1-XT con IGHx2)/(CCND1-XT,IGH-XT)x4(CCND1-XT con IGHx3)/(CCND1-XT,IGH-XT)x5(CCND1-XT con IGHx4),(TP53x1,D17Z1x2)
7	MAYO	F	83	MM	ND	37	Kappa	Sort	11;14	nuc ish(TP73x2,1q22x3),(CCND1-XT,IGH-XT)x3(CCND1-XT con IGH-XTx2)
8	MCL	M	63	MM^a^	U	73	Kappa	Fresh	11;14	nuc ish(CCND1-XTx3,IGH-XTx2)(CCND1-XT con IGHx2)/(CCND1-XTx5,IGH-XTx4)(CCND1-XT con IGHx4),(TP53x1,D17Z1x2)
9	MCL	M	69	MM	U	65	Kappa	Fresh	11;14	nuc ish(TP73,1q22,MYC,RB1,LAMP1,TP53,D17Z1)x4,(D3Z1,D7Z1,D9Z1,D15Z4)x3–4, (CCND1-XTx6,IGH-XTx7)(CCND1-XT con IGH-XTx4)
10	MCL	M	77	MM^a^	U	68	Kappa	Fresh	11;14	nuc ish(CCND1-XT,IGH-XT)x4(CCND1-XT con IGH-XTx3),(TP53x1,D17Z1x2)
11	MAYO	M	58	PCL	RR	58	Lambda	Frozen	11;14	nuc ish(CCND1-XT,IGH-XT)x4(CCND1-XT con IGH-XTx3),(RB1x1,LAMP1x2)
12	MAYO	F	77	MM	ND	50	Kappa	Frozen	11;14	nuc ish(CCND1-XTx3,IGH-XTx2)(CCND1-XT con IGH-XTx1)
13	MAYO	F	59	AL	ND	5	Lambda	CD138+	11;14	nuc ish(CCND1-XTx3),(IGH-XTx3),(CCND1-XT con IGH-XTx2)
14	MAYO	F	63	PCPD	U	5	Lambda	CD138+	11;14	nuc ish(CCND1-XTx3,IGH-XTx2)(CCND1-XT con IGH-XTx1),(RB1,LAMP1)x1
15	MAYO	F	54	MM	ND	45	Kappa	CD138+	11;14	nuc ish(CCND1-XTx2),(IGH-XTx2),(CCND1-XT con IGH-XTx1)/(CCND1-XTx2),(IGH-XTx3),(CCND1-XT con IGH-XTx1)
16	MCL	F	67	IgA gammopathy^a^	U	40	Lambda	FCP	4;14	nuc ish(TP73x2,1q22x3–4),(FGFR3,IGH)x3(FGFR3 con IGHx2),(5′MYCx3,3′MYCx2)(5′MYC con 3′MYCx2),(RB1,LAMP1)x1
17	MAYO	M	75	MM	RR	13	Kappa	Sort	4;14	nuc ish(TP73x2,1q22x3),(FGFR3,IGH)x3(FGFR3 con IGHx2),(MYCx2)(5′MYC con 3′MYCx1),(RB1,LAMP1)x1
18	MCL	M	73	MM^a^	U	34	Kappa	Sort	4;14	nuc ish(TP73x2,1q22x3),(FGFR3,IGH)x3(FGFR3 con IGHx2),(RB1,LAMP1)x1
19	MCL	M	68	Monoclonal gammopathy^a^	U	20	Lambda	Sort	4;14	nuc ish(TP73x2,1q22x3),(D3Z1,D9Z1,D15Z4)x3,(FGFR3,IGH)x3(FGFR3 con IGHx2),(RB1,LAMP1)x1
20	MCL	M	72	PCL	U	70	Kappa	Fresh	4;14	nuc ish(TP73x2,1q22x4),(FGFR3,IGH)x3(FGFR3 con IGHx2),(MYCx2)(5′MYC sep 3′MYCx1),(RB1,LAMP1)x1,(TP53x1,D17Z1x2)
21	MCL	M	42	MM^a^	U	89	Kappa	Fresh	4;14	nuc ish(TP73x2,1q22x3),(FGFR3,IGH)x3(FGFR3 con IGHx2),(MYCx4)(5′MYC sep 3′MYCx1),(RB1,LAMP1)x1,(TP53x2,D17Z1x1)
22	MAYO	M	57	MM	RR	41	Kappa	Fresh	4;14	nuc ish(TP73x2,1q22x3),(FGFR3,IGH)x3(FGFR3 con IGHx2),(5’MYCx2,3′MYCx1)(5′MYC con 3′MYCx1),(RB1,LAMP1)x1,(TP53x1,D17Z1x2)/(TP73x4,1q22x6),(D3Z1x3),(FGFR3,IGH)x4(FGFR3 con IGHx3)/(FGFR3,IGH)x5(FGFR3 con IGHx4),(D7Z1,D9Z1,D15Z4)x3–4,(5′MYCx4,3′MYCx2)(5’MYC con 3’MYCx2),(CCND1-XTx4),(RB1,LAMP1)x2,(TP53x2,D17Z1x4)
23	MAYO	M	78	MM	RR	77	Lambda	Frozen	4;14	nuc ish(TP73x2,1q22x3),(FGFR3,IGH)x2(FGFR3 con IGHx1),(RB1,LAMP1)x1,(TP53x1,D17Z1x2)
24	MCL	M	63	MM^a^	U	13	Kappa	Sort	14;16	nuc ish(TP73x2,1q22x3),(D9Z1,RB1,LAMP1)x1,(IGHx4,MAFx3)(IGH con MAFx2)
25	MAYO	F	60	MM	ND	31	Kappa	Sort	14;16	nuc ish(TP73x2,1q22x3),(RB1,LAMP1)x1,(IGHx3,MAFx2)(IGH con MAFx2)
26	MAYO	F	80	MM	RR	25	Kappa	CD138+	14;16	nuc ish(D3Z1,D9Z1,p53,D17Z1)x3/(Rb1,LAMP1,D15Z4)x1/(IGHx2),(c-MAFx3),(IGH con c-MAFx1)
27	MAYO	F	67	MM	ND	60	Kappa	CD138+	14;16	nuc ish(IGHx3),(MAFx3),(IGH con MAFx2)
28	MCL	M	75	MM^a^	U	27	Kappa	FCP	14;20	nuc ish(TP73x4,1q22x6–8),(5′MYC,3′MYC)x4(5′MYC con 3′MYCx2),(RB1,LAMP1)x2,(IGHx6,MAFBx4)(IGH con MAFBx2),(TP53,D17Z1)x4
29	MCL	M	88	MM^a^	U	56	Kappa	FCP	14;20	nuc ish(MAFBx2,IGHx3)(MAFB con IGHx2),(RB1,LAMP1)x1
30	MCL	F	65	R/O MM^a^	U	63	Kappa	Fresh	14;20	nuc ish(MYCx2)(5′MYC sep 3′MYCx1),(RB1,LAMP1)x1,(IGH,MAFB)x3(IGH con MAFBx2)/(IGHx4,MAFBx3)(IGH con MAFBx2)
31	MAYO	F	74	MM	ND	5	Kappa	Fresh	14;20	ish(TP73x3,1q22x6),(D3Z1,D9Z1,D15Z4,TP53,D17Z1)x4,(D7Z1x5),(MYCx3–4),(CCND1-XTx4–5),(RB1,LAMP1)x2,(IGHx3,MAFBx4)(IGH con MAFBx1)
32	MCL	M	74	MM^a^	U	N/A	N/A	FCP	6;14	nuc ish(TP73,1q22)x3,(CCND3x2,IGHx1)(CCND3 con IGHx1),(RB1,LAMP1)x1,(TP53x1,D17Z1x2)
33	MCL	M	51	R/O MM, hypercalcemia, renal failure^a^	U	N/A	N/A	FCP	6;14	84,XX,der(Y;16)(q10;p10)x2,i(1)(q10)x2,–2,–4,–4,t(6;14)(p21.1;q32)x2,−8,t(8;14) (q24.1;q32),−10,−11,der(14)t(8;14)^6^/46,XY^14^ nuc ish,(CCND3x2,IGHx1)(CCND3 con IGHx1) only *CCND3/IGH* FISH performed on FCP sample
34	MCL	F	58	MM^a^	U	21	Kappa	FCP	Hyper + IGH sep	nuc ish(TP73x2,1q22x3),(D7Z1,MYC)x3,(RB1,LAMP1,D15Z4)x1,(3′IGHx2,5’IGHx1)(3′IGH con 5′IGHx1)/(IGHx2)(3’IGH sep 5′IGHx1),(TP53x1,D17Z1x2)
35	MCL	F	54	MM^a^	U	31	Kappa	FCP	Hyper + IGH sep	nuc ish(D3Z1,D9Z1,D15Z4)x3,(FGFR3,CCND3,MAF,MAFB)x1,(MYCx2)(5′MYC sep 3′MYCx1),(CCND1-XTx4–5),(3′IGHx3,5′IGHx2)(3′IGH con 5′IGHx2)/(IGHx3)(3′IGH sep 5′IGHx2)
36	MAYO	M	82	MM	RR	7	Lambda	Sort	Hyper + IGH sep	nuc ish(TP73x2,1q22x3),(CCND3,D7Z1,D9Z1)x3,(3′IGHx2,5′IGHx1)(3′IGH con 5′IGHx1)
37	MAYO	M	61	MM	RR	27	Kappa	Sort	Hyper + IGH sep	nuc ish(5′MYCx2,3′MYCx1)(5′MYC con3′MYCx1)/(D9Z1x3–4)/(CCND1-XTx3)/(3′IGHx2,5′IGHx1)(3′IGH con 5′IGHx1)/(3′IGHx3,5′IGHx2)(3′IGH con 5′IGHx2)
38	MAYO	F	58	MM	RR	11	Lambda	Sort	Hyper + IGH sep	nuc ish(TP73x2,1q22x4–5),(D3Z1,D7Z1,D9Z1,CCND1-XT,TP53,D17Z1)x3,(RB1x1,LAMP1x2),(3′IGHx2,5’IGHx1)(3′IGH con 5’IGHx1)
39	MCL	M	64	MM	U	20	Kappa	Fresh	Hyper + IGH sep	nuc ish(TP73x2,1q22x3),(MYC,D9Z1,CCND1-XT,D15Z4)x3,(3′IGHx3,5′IGHx2)(3′IGH con 5′IGHx2)
40	MCL	M	80	MM	U	81	Lambda	Fresh	Hyper + IGH sep	nuc ish(TP73x2,1q22x3),(FGFR3,MAF,MAFB)x1,(CCND3,D9Z1,D15Z4)x3,(3′IGHx3,5′IGHx2)(3′IGH con 5′IGHx2),(TP53x1,D17Z1x2)
41	MCL	M	60	MM^a^	U	98	Kappa	Fresh	Hyper + IGH sep	nuc ish(TP73x3,1q22x2),(D3Z1,CCND3,CCND1-XT,D15Z4)x3,(MYCx2)(5′MYC sep 3′MYCx1),(RB1,LAMP1,MAF)x1,(3′IGHx4–5,5′IGHx4)(3’IGH con 5′IGHx3),(TP53x1,D17Z1x2)
42	MAYO	M	70	MM	RR	19	Kappa	Sort	Hyper + IGH sep	nuc ish(D3Z1,D7Z1,D9Z1,D15Z4)x3,(5′MYCx3,3′MYCx2)(5′MYC con 3′MYCx2),(CCND1-XT amp),(3′IGHx3,5′IGHx2)(3′IGH con 5′IGHx2)
43	MAYO	F	56	PCPD	U	4	Indeterminate	Sort	Hyper	nuc ish(CCND1-XTx3),(RB1,LAMP1)x1
44	MAYO	M	67	MM	RR	12	Kappa	Sort	Hyper	nuc ish(CCND1-XTx3),(RB1,LAMP1,IGH,TP53,D17Z1)x1
45	MCL	M	76	MM^a^	U	14	Kappa	Sort	Hyper	nuc ish(TP73x2,1q22x3),(D3Z1,D9Z1,CCND1-XT)x3,(MYCx2)(5′MYC sep 3′MYCx1),(D15Z4x3–4)
46	MAYO	M	66	MM	ND	19	Kappa	Sort	Hyper	nuc ish(TP73x2,1q22x3),(D3Z1,MYC,CCND1-XT,D15Z4)x3,(D9Z1x3–4)
47	MAYO	M	72	MM	ND	10	Kappa	Sort	Hyper	nuc ish(TP73x2,1q22x3),(D3Z1,MYC)x3,(D7Z1,D9Z1)x3–4,(RB1,LAMP1,IGH,D15Z4)x1
48	MCL	F	77	Anemia, coagulation defect^a^	U	10	Kappa	Sort	Hyper	nuc ish(TP73x2,1q22x3),(D7Z1,D9Z1,D15Z4)x3,(RB1x1,LAMP1x2)
49	MCL	M	73	R/O MM^a^	U	35	Kappa	Sort	Hyper	nuc ish(TP73x2,1q22x3),(D3Z1,D7Z1,D9Z1,CCND1-XT)x3,(D15Z4x3–4)
50	MCL	F	64	MM^a^	U	48	Kappa	Fresh	Hyper	nuc ish(CCND1-XTx4),(IGHx3),(MAFx1)
51	MAYO	F	49	MM	RR	84	Lambda	Fresh	Hyper	nuc ish(TP73x2,1q22x3–4),(D3Z1,D9Z1)x3,(5′MYCx4–5,3′MYCx1)(5’MYC con 3′MYCx1),(CCND1-XT,D15Z4)x3–4,(TP53x1,D17Z1x2)
52	MCL	F	75	PCPD^a^	U	73	Lambda	Fresh	Hyper	nuc ish(D3Z1,D7Z1,D9Z1)x3,(RB1,LAMP1,IGH)x1,(TP53x1,D17Z1x2)
53	MCL	F	66	MM^a^	U	52	Kappa	Fresh	Hyper	nuc ish(D3Z1,D9Z1,CCND1-XT,D15Z4)x3
54	MCL	M	65	MM^a^	U	52	Lambda	Fresh	Hyper	nuc ish(TP73x2,1q22x3–4),(D3Z1,D7Z1,D9Z1,IGH,D15Z4,TP53,D17Z1)x3,(CCND1-XTx3–4),(RB1,LAMP1)x1/(RB1x2,LAMP1x1)
55	MCL	M	57	Solitary plasmacytoma	U	99	Kappa	Fresh	Hyper	nuc ish(TP73x2,1q22x3),(D3Z1,D9Z1)x3,(CCND1-XT,D15Z4)x3–4,(RB1x1,LAMP1x2)
56	MCL	F	56	MM^a^	U	34	Kappa	Fresh	Hyper	nuc ish(D3Z1,D7Z1,CCND1-XT)x3,(D9Z1,D15Z4)x3–4
57	MAYO	M	61	MM	RR	55	Kappa	Frozen	Hyper	nuc ish(TP73x2,1q22x3),(D3Z1,D7Z1,D15Z4)x3,(D9Z1)x3–4,(RB1x1,LAMP1x2)
58	MAYO	M	59	MM	RR	79	Indeterminate	Frozen	Hyper	nuc ish(TP73x2,1q22x3),(D3Z1,CCND1-XT)x3
59	MAYO	M	74	MM	RR	60	Kappa	CD138+	Hyper	nuc ish(D3Z1,D7Z1,D9Z1,D15Z4)x3
60	MAYO	M	53	MM	ND	90	Kappa	CD138+	Hyper	nuc ish(D3Z1,D9Z1,CCND1-XT,D15Z4),(IGH)x1
61	MAYO	F	61	MM	RR	25	Kappa	CD138+	Hyper	nuc ish(D3Z1,D7Z1,D9Z1,CCND1)x3/(Rb1,LAMP1)x1/(D15Z4x3–4) nuc ish(TP73x2,CKS1Bx3),(MYCx3)
62	MAYO	M	48	MM	RR	75	Lambda	CD138+	Hyper	nuc ish(D3Z1,D9Z1,CCND1-XT,D15Z4)x3/(IGHx1)
63	MAYO	F	61	MM	RR	5	Kappa	CD138+	Hyper	nuc ish(D9Z1,CCND1-XT,D15Z4)x3
64	MAYO	F	48	MM	RR	70	Kappa	CD138+	Hyper	nuc ish(D3Z1,D7Z1,CCND1-XT)x3/(D9Z1,D15Z4)x3–4
65	MAYO	F	66	MM	RR	25	Lambda	CD138+	Hyper	nuc ish(CCND1-XT)x3
66	MCL	F	70	MM	U	39	Lambda	Sort	Tetraploid	nuc ish(TP73x2,1q22x4–6),(D3Z1,TP53,D17Z1)x4,(D7Z1,MYC,D9Z1,CCND1-XT,RB1,LAMP1,IGH,D15Z4)x3–4
67	MCL	M	74	Gammopathy^a^	U	36	Lambda	Sort	Monosomy 13/14	nuc ish(TP73x1,1q22x2),(RB1,LAMP1,IGH)x1
68	MAYO	M	58	MM	ND	15	Lambda	CD138+	Monosomy 15	nuc ish(D15Z4x1)
69	MAYO	F	62	AL	RR	5	Lambda	CD138+	Monosomy 15	nuc ish(D15Z4x1)
70	MAYO	F	68	AL	RR	9	Kappa	CD138+	Normal	Normal

Cohort of 70 patients evaluated. *MCL* Mayo Clinic Laboratories, *F* female, *M* male, *Dx* diagnosis, *RFR* reason for referral, *AL* amyloidosis, *ND* newly diagnosis, *RR* relapsed/refractory, *U* unknown status. ^a^Indicates only RFR is available. % PC from flow cytometry

Only abnormal FISH results are indicated in the ISCN

**Table 2:** Patient characteristics

Total (*N* = 70)	
Characteristic	*N* (%)
Sex	
Male	40 (57.1)
Female	30 (42.9)
Age	
Median	66 years
Range	42–88 years
40–49	4 (5.7)
50–59	14 (20.0)
60–69	26 (37.1)
70–79	21 (30.0)
80–89	5 (7.1)
Diagnosis or RFR	
MM, PCN diagnosis or RFR	57 (81.4)
Amyloidosis	3 (4.3)
Plasma cell leukemia	2 (2.9)
Plasma cell proliferative disorder	3 (4.3)
Other	5 (7.1)
Site	
Mayo Clinic-local	37 (52.9)
Mayo Clinic Laboratories-outside	33 (47.1)
PC percentage (N = 68)	
Median	35.5%
Range	4–99%
4–19	19 (27.9)
20–39	19 (27.9)
40–59	11 (16.2)
60–79	12 (17.6)
80–99	7 (10.3)
Sample type	
No enrichment	31 (44.3)
Fixed cell pellet (FCP)	8 (11.4) median PCs (35.5)
Fresh sample	18 (25.7) median PCs (66.5)
Frozen sample	5 (7.1) median PCs (58.0)
Enrichment	39 (55.7)
Flow sorting	24 (34.3) median PCs (19.5)
CD138+ magnetic	15 (21.4) median PCs (25.0)
Light chain	
Kappa	46 (65.7)
Lambda	20 (28.6)
Indeterminate or unknown	4 (5.7)
Primary cytogenetic abnormality	
t(11;14)	15 (21.4)
t(4;14)	8 (11.4)
t(14;16)	4 (5.7)
t(14;20)	4 (5.7)
t(6;14)	2 (2.9)
Hyperdiploid only	23 (32.9)
Hyperdiploid with an unknown IGH rearrangement	9 (12.9)
Tetraploid without primary abnormality	1 (1.4)
Monosomy 13/14 alone	1 (1.4)
Monosomy 15 alone	2 (2.9)
Normal	1 (1.4)
Conventional chromosome study	
Not performed	28 (40.0)
Performed	42 (60.0)
Normal or loss of Y	27 (38.6 of total, 64.3 of performed)
Abnormal with PC abnormalities	14 (20.0 of total, 33.3 of performed)
Abnormal with non-PC abnormalities	1 (1.4 of total, 2.4 of performed)

Patient characteristics of the 70 patients within the cohort evaluated

### MM abnormalities identified by FISH

Recurrent primary MM cytogenetic abnormalities identified by FISH in samples 1–65 were t(11;14) (21.4%), t(4;14) (11.4%), t(14;16) (5.7%), t(14;20) (5.7%), t(6;14) (2.9%), and hyperdiploidy (45.7%) either without an IGH rearrangement (32.9%) or with an IGH rearrangement that did not involve *CCND1*, *FGFR3*, *MAF*, *MAFB* or *CCND3* (12.9%) (Tables 1, 2). Five samples (cases 66–70) had undefined primary abnormalities including one case of tetrapoloidy with a relative 1q gain, one case with monosomies 13 and 14, two cases with monosomy 15 by FISH and a single case with normal FISH results in a patient with a diagnosis of amyloidosis (Tables 1, 2). Conventional chromosome studies were performed on 42 (60.0%) cases and an abnormal PC clone was identified in 33.3% of the cases with chromosome studies performed (Supplemental Table 3).

We have previously determined tumor content requirements for MPseq requiring 10% tumor for the detection of structural rearrangements and 25% tumor for the detection of CNAs^18^. Since variable and sometimes low clonal PC percentages can be identified in the BM aspirates of patients with NDMM^31^, we performed two enrichment strategies for samples with low PCs including magnetic enrichment or flow sorting. For some samples, no enrichment was performed. Thirty-nine (55.7%) samples with a median 23.0% PCs were subjected to either flow sorting (*N* = 24) or CD138 + magnetic bead for PC enrichment (*N* = 15). For the remaining 31 samples (44.3%) with a median 58% PC, no PC enrichment was performed.

### Identification of recurrent, primary cytogenetic abnormalities using MPseq

**Fig. 1:**
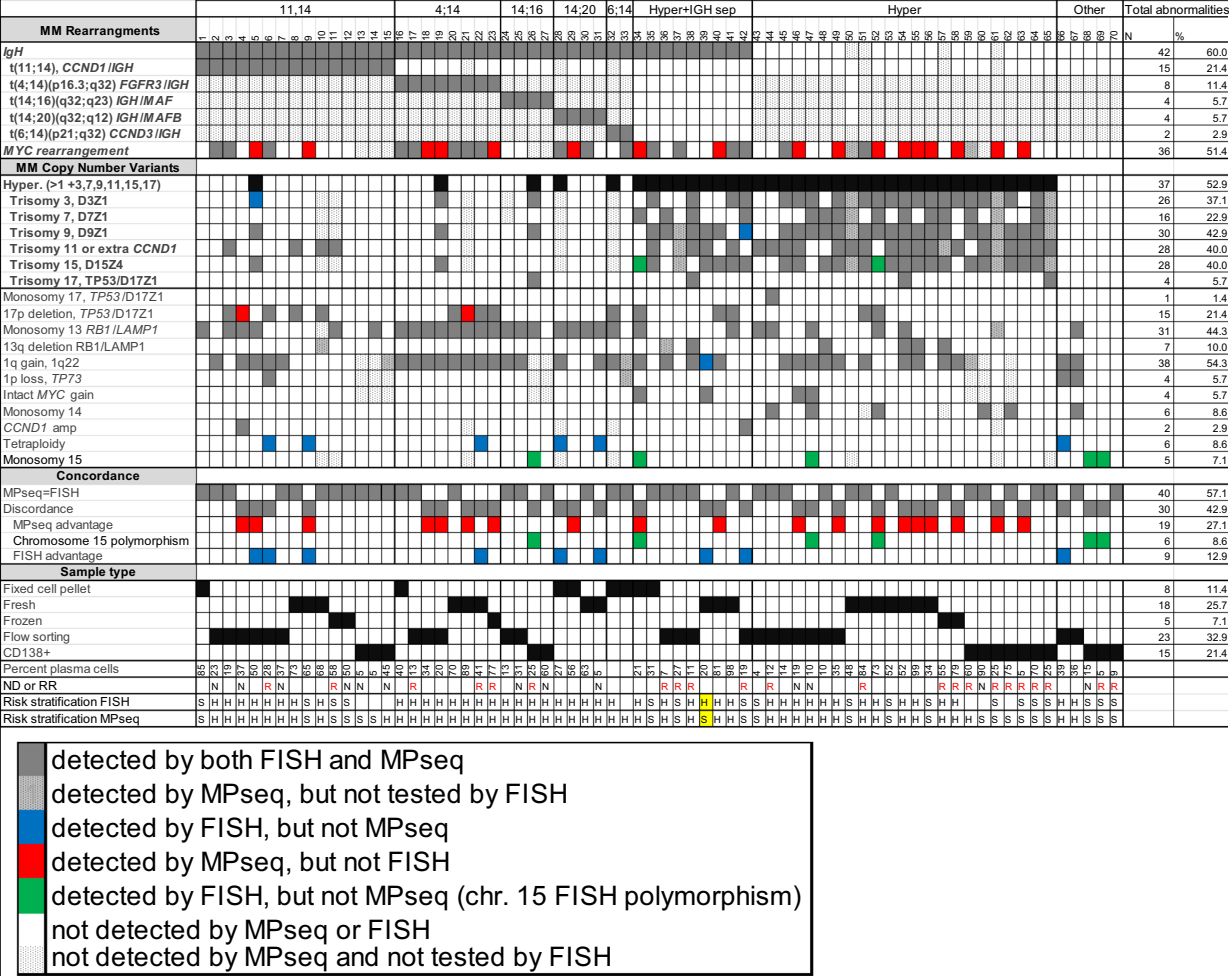
Concordance between MPseq and FISH. In bold indicates primary cytogenetic abnormalities. Cytogenetic risk applied to all cases: H: high and S:standard. ND: Newly diagnosed, RR: relapsed/refractory. For case 33, there was no FISH for MYC BAP, but detection of t(8;14) and t(6;14) was achieved using chromosome studies and *CCND3/IGH* rearrangement confirmed by FISH. For case 37, there was a history of trisomies 9,11,15, and IGH separation in an older sample. For cases 61 and 65 there was evidence of hyperdiploidy by FISH in older samples. Highlighted in yellow is a single case with a difference in cytogenetic risk between MPseq and FISH.

To determine the accuracy of MPseq in comparison to our PCN FISH panel (Supplemental Table 1) in the detection of recurrent, primary MM abnormalities (IGH rearrangement and/or hyperdiploidy), we analyzed DNA extracted from either a fixed cell pellet (FCP) from a chromosome study (*n* = 8), from fresh (*n* = 18) or frozen (*n* = 5) BM aspirates or from fresh BM specimens that had been flow sorted (*n* = 24) or subjected to CD138 + magnetic enrichment (*n* = 15) (Supplemental Fig. 1, Tables 1, 2). For samples 1–65, MPseq confirmed the primary abnormality identified by FISH in each case demonstrating 100% concordance between both assays for the classification of recurrent, primary cytogenetic abnormalities (Fig. 1). For those cases without evidence of a recurrent, primary abnormality (samples 66–69), MPseq did not identify tetraploidy in case 66 and monosomy 15 in cases 68–69, but identified monosomies 13 and 14 in case 67 and confirmed no recurrent abnormality in case 70 with normal FISH results. As a negative control, no recurrent primary MM abnormalities (MM specific IGH rearrangements and/or hyperdiploidy with gains of odd numbered chromosomes) were identified by MPseq in a previously described cohort of 88 patients with a reason for referral of acute myeloid leukemia (data not shown)^18^.

### Comparison of MPseq to FISH for detection of recurrent, secondary abnormalities

**Fig. 2:**
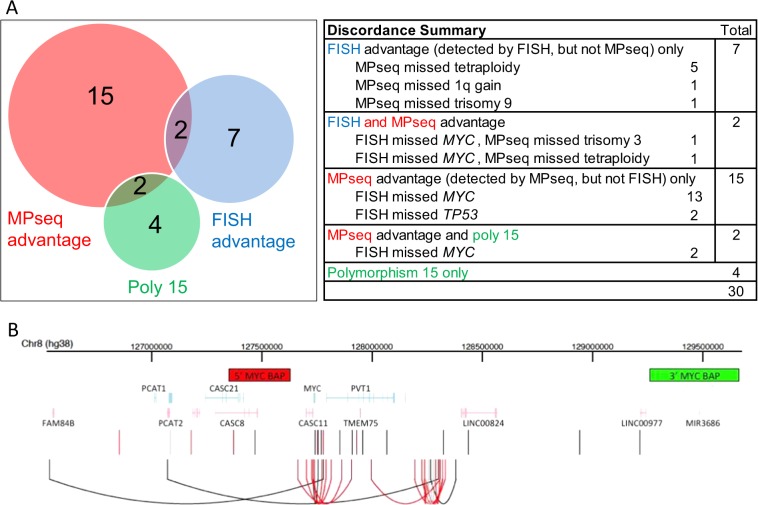
Discordance summary and *MYC* breakpoint locations. **a** Total number of cases with evidence of MPseq advantage, FISH advantage and polymorphism of chromosome 15. **b** The location of breakpoints in the *MYC* locus across all cases are depicted as vertical lines (black if the *MYC* alteration was detected by FISH, light gray if it was not tested by FISH, and red if it was undetected by FISH). In cases where multiple breakpoints were found in the *MYC* locus, the lines are connected by an arc. The locations of the *MYC* BAP probes used for FISH detection are shown at the top (5′ in red, 3′ in green) and gene locations are shown in the middle (forward strand in light blue, reverse strand in pink).

For each primary and secondary abnormality that was identified by either MPseq or FISH, 40 cases (57.1%) displayed concordance between FISH and MPseq. Thirty cases (42.9%) displayed at least one instance of discordance between FISH and MPseq (Figs. 1, 2a). Nine of these 30 discordant cases had abnormalities detected by FISH that went undetected by MPseq (Figs. 1, 2a, “FISH advantage”). Of these nine cases, six had a tetraploid clone that was not detectable by MPseq and in three cases MPseq failed to detect CNAs that were identified by FISH (trisomy 3, trisomy 9 and 1q gain). In six cases, FISH identified a CNA involving chromosome 15 that was not confirmed by MPseq. These abnormalities included monosomy 15 identified by FISH without evidence of monosomy 15 by MPseq (cases 26, 47, 68, 69), or one (case 34) or two (case 52) copies of chromosomes 15 identified by FISH in cases with trisomy 15 identified by MPseq (Figs. 1, 2a). In contrast, 19 of the 30 discordant cases had abnormalities detected by MPseq that went undetected by FISH (Figs. 1, 2a, “MPseq advantage”). Of these 19 cases, 17 were *MYC* rearrangements and two were 17p deletions (cases 4 and 21), including a 17p translocation involving the *TP53* gene in one case (Figs. 1, 2a).

### Increased detection rate of *MYC* rearrangements by MPseq

From 70 total cases, we identified 36 cases (51.4%) that displayed a *MYC* rearrangement by MPseq (Fig. 1). Of these 36 cases, 34 had FISH data evaluating the *MYC* locus. Seventeen cases (50.0% of *MYC* abnormal group with FISH results) displayed a false negative *MYC* FISH result where a *MYC* rearrangement was identified by MPseq, but was negative by FISH (Fig. 1). The most common partner gene/enhancer segment identified were IGH (*n* = 7), *FAM46C* (n = 5), IGK (*n* = 4), *NSMCE2* (*n* = 4), *TXNDC5* (*n* = 4) and *IGL* (*n* = 4) (Table 3). Of the 36 *MYC* rearrangements, multiple mechanisms resulting in positioning of *MYC* near enhancer sequences including small insertions, inversions, simple, balanced or complex translocations were identified (Table 3). The most common method of rearrangement identified in 15 cases included a small insertion of enhancer sequences near the *MYC* gene or, alternately, the insertion of *MYC* near enhancer sequences. These insertions typically involve the duplication of genetic material of similar size at both the source location and the insertion location, whereby the source DNA is inserted between flanking duplications at the insertion location^32^ (Fig. 2b). Thirteen of these 15 insertion cases co-occurred with hyperdiploidy (hyperdiploidy only or hyperdiploidy with IGH separation) and two of these cases were identified by FISH studies. Of the 17 *MYC* cases that were missed by FISH, 11 represented these small insertions (Table 3).

**Table 3:**
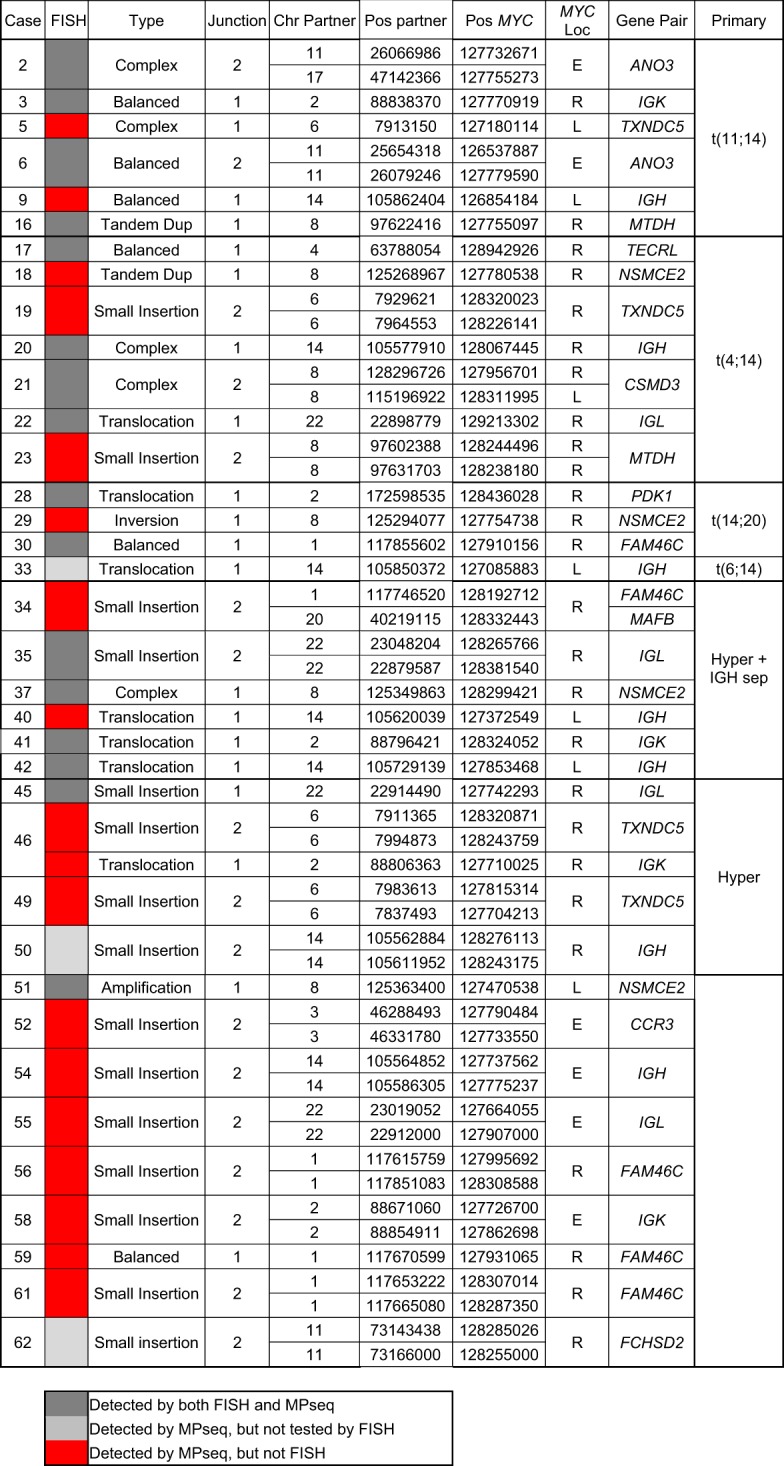
Genetic information of secondary alterations involving *MYC*.

For each case where a secondary alteration involving *MYC* was found, the relevant genomic information is provided for the junction(s). The case column is the case number. The FISH column indicates whether or not the *MYC* FISH test detected the secondary alteration (dark gray–detected by both FISH, light gray–detected by MPseq but not tested by FISH and red–detected by MPseq only). The type column is the type of alteration involved with *MYC* classified as either a balanced event, a tandem duplication, a translocation, an inversion, part of an amplification, part of a small insertion motif, a complex event, or ND where it was not possible to definitively classify the alteration. The Junction column is the number of junctions involved directly in the alteration, either 1 or 2. The Chr Partner and Pos Partner columns are the chromosome and position location (GRCh38) of the partner breakpoints that are part of alteration. The Pos *MYC* and *MYC* Loc columns give the position of the breakpoint in the *MYC* locus and whether the alteration is to the left, right, or encompassing (L, R, or E) the *MYC* gene, respectively. The Gene Pair column is the gene that is found at or near the partner breakpoint location. The Primary column is the primary alteration for the case

### Detection of additional genomic alterations by MPseq that are not evaluated by FISH

**Fig. 3:**
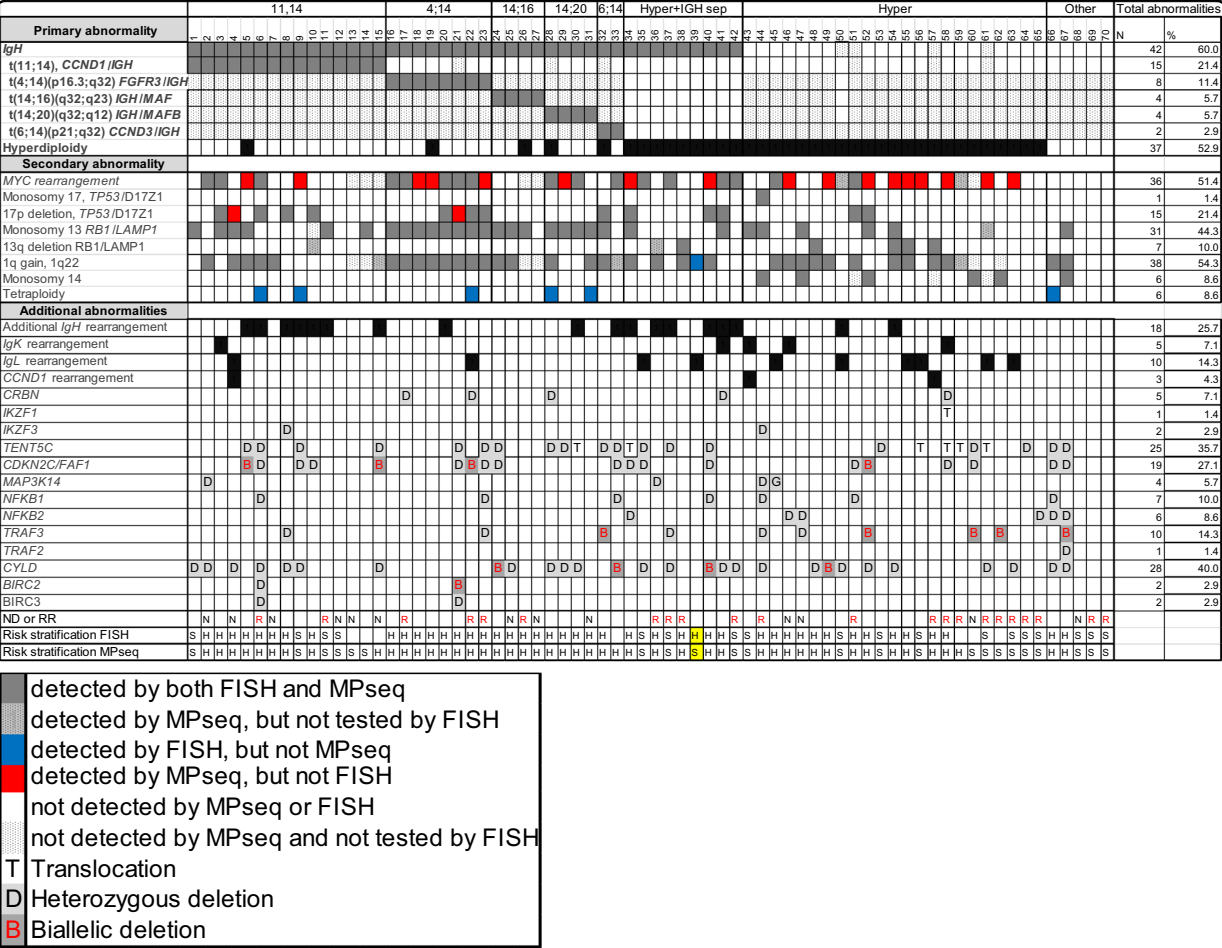
Detection of additional abnormalities by MPseq in relation to each primary and secondary abnormality. In bold indicates primary abnormalities. Cytogenetic risk applied to all cases: H: high and S:standard. ND: Newly diagnosed, RR: relapsed/refractory.

We next evaluated for the presence of rearrangements involving non-recurrent IGH MM partners (excluding *CCND1*, *FGFR3*, *MAF*, *MAFB* or *CCND3*) and the IGK and IGL loci by MPSeq. There were 19 additional IGH rearrangements identified in 18 cases (25.7% of cohort) with partner chromosomes at 8q24.21 (*MYC*) (*n* = 7) as the only recurrent rearrangement (Table 4, Fig. 3). Of the nine cases classified as “hyperdiploidy with IGH separation”, an IGH partner was identified in six cases, while the other three cases had a loss within the IGH locus. Two cases (cases 50 and 54) classified as hyperdiploidy without an IGH rearrangement to one of the common partner chromosomes had the “small insertion” type of *MYC/IGH* rearrangement (Tables 3, 4, Fig. 3). There were three cases with a *CCND1* rearrangement to a locus other than IGH (IGK/*CCND1* in case 43, IGL/*CCND1* in case 4 and *BRINP3*/*CCND1* in case 57) that had additional copies of *CCND1* observed by FISH in case 4 and 43. FISH for *CCND1* was not performed in case 57 and in case 4, the signal pattern for *CCND1* was scored as amplification (Table 4, Figs. 1, 3).

**Table 4:** IGH, IGK, and IGL partner genes.

Case	IGH partner chromosome	Putative gene target	Primary abnormality
5	14q24.3	*BATF*	11;14
6	19p13.2	*TYK2*	11;14
8	11q14.1	*RAB39*	11;14
9	1p35.3	*PTPRU*	11;14
9	8q24.21	*MYC*	11;14
10	20q11.21	*COMMD7*	11;14
11	22q13.1	*POLR2F*	11;14
15	2p24.3	*MYCN*	11;14
20	8q24.21	*MYC*	4;14
30	5p15.33	*TERT*	14;20
33	8q24.21	*MYC*	6;14
34	7q32.1	*Unknown*	Hyper + IGH sep
36	Xq32.33	*MTMR1*	Hyper + IGH sep
37	14q24.3	*DPF3*	Hyper + IGH sep
40	8q24.21	*MYC*	Hyper + IGH sep
41	9p13.2	*PAX5*	Hyper + IGH sep
42	8q24.21	*MYC*	Hyper + IGH sep
50	8q24.21	*MYC*	Hyper
54	8q24.21	*MYC*	Hyper

Case	IGK partner chromosome	Putative gene target	Primary abnormality
3	8q24.21	*MYC*	11;14
41	8q24.21	*MYC*	Hyper + IGH sep
43	11q13.3	*CCND1*	Hyper
46	8q24.21	*MYC*	Hyper
58	8q24.21	*MYC*	Hyper

Case	IGL partner chromosome	Putative gene target	Primary abnormality
4	11q13.3	*CCND1*	11;14
22	8q24.21	*MYC*	4;14
35	8q24.21	*MYC*	Hyper + IGH sep
39	3q26.2	*MECOM*	Hyper + IGH sep
45	8q24.21	*MYC*	Hyper
50	17q25.1	*GRB2*	Hyper
55	8q24.21	*MYC*	Hyper
56	8q24.22	*ST3GAL1/NDRG1*	Hyper
61	8q24.22	*ST3GAL1/NDRG1*	Hyper
63	8q24.22	*ST3GAL1/NDRG1*	Hyper

Partner genes associated with IGH, IGK, and IGL showing cytogenetic location and putative target genes. Hyper: Hyperdiploidy only. Hyper+IGH sep: Hyperdiploidy with IGH separation

There were five cases with IGK rearrangements (7.1% of cohort) mainly with partner chromosome 8q24.21 (*MYC*) (n = 4) and a single case with partner chromosome at 11q13.3 (*CCND1*) (Table 4, Fig. 3). In addition, 10 cases (14.3% of cohort) had IGL rearrangements with partner chromosomes at 8q24.21 (*MYC*) (n = 4), 11q13.3 (*CCND1*) (n = 1), 8q24.22 (n = 3) (putative target *ST3GAL1/NDRG1)*, 3q26.2 (*MECOM*) (n = 1) and 17q25.1 (*GRB2*) (n = 1) (Table 4, Fig. 3). Of these 15 cases with either an IGK or IGL rearrangement, 12 (80.0%) co-occurred with hyperdiploidy (hyperdiploidy only or hyperdiploidy with IGH separation).

We explored alterations in additional genes contributing to dysregulation of multiple pathways such as WNT or NF-kB signaling including genes *CYLD* at 16q12.1, *BIRC2* and *BIRC3* at 11q22,2, *NFKB1* at 4q24*, NFKB2* at 10q24.32, *TRAF2* at 9q34.3*, TRAF3 at 14q32.32* and *MAP3K14*/*NIK* at 17q21.31 or other tumor suppressor genes such as *CDKN2C* (p18) at 1p32.3 or *TENT5C/FAM46C* at 1p12^33,34^ (Table 5, Fig. 3). Twenty-five cases (35.7%) had an alteration in *TENT5C/FAM46C* with 6 cases with translocations (five of these to *MYC*) and 19 cases had a heterozygous deletion of *TENT5C/FAM46C* ranging in size from 3.4 Mb to 120 Mb. Nineteen cases (27.1%) had alterations in *CDKN2C and/*or *FAF1*. Fourteen were heterozygous deletions involving *CDKN2C* ranging in size from 587 Kb to 120 Mb, four cases had focal biallelic *CDKN2C and FAF1* deletions (Supplemental Fig. 2A, case #5) and one case had a heterozygous 655 Kb *FAF1* deletion without a *CDKN2C* deletion (Table 5, Fig. 3). Ten cases had deletions of *TRAF3* with 5 as heterozygous deletions and five as biallelic deletions (Supplemental Fig. 2B, case #62) and a single case had a 92.9 Kb heterozygous deletion of *TRAF2* (Table 5, Fig. 3). Twenty-eight cases had deletions of *CYLD* (40% of cohort) with 24 cases having heterozygous deletions ranging in size from 634 Kb to 90.3 Mb with the majority representing large 16q deletions and four cases will smaller biallelic deletions (Table 5, Fig. 3, Supplemental Fig. 2C, case #40). Additional alterations in *MAP3K14* were observed in four cases (three as heterozygous deletions and one as a 735 Kb gain), heterozygous deletion of *NFKB1* in seven cases, heterozygous deletion of *NFKB2* in 6 cases and a heterozygous and homozygous *BIRC2* and *BIRC3* deletions in two separate cases (Table 5, Fig. 3).

**Table 5:** Abnormalities of additional genes of clinical significance

Case	CYLD	Location	Breakpoints	Size (bp)	Primary
1	HD	16p13.3–16q24.3	0–90338345	90338345	11;14
2	HD	16q11.2–16q24.3	46454000–90338000	43884000	11;14
4	HD	16q11.2–16q24.3	46454000–90338000	43884000	11;14
6	HD	16q11.2–16q24.3	46454000–90338000	43884000	11;14
8	HD	16q11.2–16q24.3	46454000–90338000	43884000	11;14
9	HD	16q21.1–16q24.3	50093000–89129000	39036000	11;14
15	HD	16q11.2–16q24.3	46454000–90338000	43884000	11;14
24	**BD**	16q12.1–16q12.1	50232040–50913020	680980	14;16
25	HD	16q11.2–16q24.3	46454000–90338000	43884000	14;16
28	HD	16q11.2–16q24.3	46454000–90338000	43884000	14;20
29	HD	16q11.2–16q24.3	46454000–90338000	43884000	14;20
30	HD	16q11.2–16q24.3	46454000–90338000	43884000	14;20
33	**BD**	16q12.1–16q12.1	50777028–50812200	35172	6;14
35	HD	16q11.2–16q24.3	46454000–90338000	43884000	Hyper + IGH sep
37	HD	16q11.2–16q24.3	46454000–90338000	43884000	Hyper + IGH sep
40	**BD**	16q12.1–16q12.2	50376741–52630833	2254092	Hyper + IGH sep
41	HD	16q11.2–16q24.3	46454000–90338000	43884000	Hyper + IGH sep
42	HD	16q12.1–16q12.1	50193000–50827000	634000	Hyper + IGH sep
44	HD	16q11.2–16q24.3	46454000–90338000	43884000	Hyper
48	HD	16q12.1–16q12.2	50123000–55838000	5715000	Hyper
49	**BD**	16q12.1–1612.1	50290162–51082053	791891	Hyper
50	HD	16q11.2–16q24.3	46454000–90338000	43884000	Hyper
52	HD	16q11.2–16q24.3	46454000–90338000	43884000	Hyper
54	HD	16q11.2–16q24.3	46454000–90338000	43884000	Hyper
61	HD	16q11.2–16q24.3	46454000–90338000	43884000	Hyper
63	HD	16q11.2–16q24.3	46454000–90338000	43884000	Hyper
66	HD	16q11.2–16q24.3	46454000–90338000	43884000	Tetraploid
67	HD	16p13.3–16q24.3	0–90338345	90338345	Monosomy 13/14

Case	BIRC2 and BIRC3	Location	Breakpoints	Size (bp)	Primary
6	HD	11q14.1–11q22.3	79621665–108999346	29377681	11;14
21	**BD**	11q22.1–11q22.2	101044665–102389301	1344636	4;14

Case	TENT5C/FAM46C	Location	Breakpoints	Size (bp)	Primary
5	HD	1p22.3–1p12	87833886–119707445	31873559	11;14
6	HD	1p36.33–1p12	1–119990000	119989999	11;14
9	HD	1p35.3–1p12	29234000–119991000	90757000	11;14
15	HD	1p32.3–1p12	51107000–119761000	68654000	11;14
21	HD	1p31.1–1p12	77992000–119733000	41741000	4;14
23	HD	1p34.2–1p12	42342000–121700000	79358000	4;14
24	HD	1p13.3–1p12	110882000–120028000	9146000	14;16
28	HD	1p32.1–1p12	58536000–119985000	61449000	14;20
29	HD	1p31.1–1p12	75850000–118934000	43084000	14;20
30	Translocation to MYC	1p12	117855602	N/A	14;20
32	HD	1p31.1–1p12	70504000–119991000	49487000	6;14
33	HD	1p36.33–1p12	1–119982000	119981999	6;14
34	Translocation to MYC and MAFB	1p12	117611301;117746520	N/A	Hyper + IGH sep
35	HD	1p33–1p12	49064000–119989000	70925000	Hyper + IGH sep
37	HD	1p22.2–1p12	89819000–118483000	28664000	Hyper + IGH sep
40	HD	1p13.1–1p12	116616000–119990000	3374000	Hyper + IGH sep
53	HD	1p31.1–1p12	77694018–119983000	42288982	Hyper
56	Translocation to MYC	1p12	117615759;117851083	N/A	Hyper
58	Translocation to IL16	1p12	117592488;117745524	N/A	Hyper
59	Translocation to MYC	1p12	117670599	N/A	Hyper
60	HD	1p22.1–1p12	92935000–119981000	27046000	Hyper
61	Translocation to MYC	1p12	117653222;117665080	N/A	Hyper
64	HD	1p31.3–1p12	67860000–118597000	50737000	Hyper
66	HD	1p36.33–1p12	1–119990000	119989999	Tetraploid
67	HD	1p36.33–1p12	1–119991000	119990999	Monosomy 13/14

Case	CDKN2C and FAF1	Location	Breakpoints	Size (bp)	Primary
5	**BD**	1p32.3–1p32.3	50884258–51012825	128567	11;14
6	HD	1p36.33–1p12	1–119990000	119989999	11;14
9	HD	1p35.3–1p12	29234000–119991000	90757000	11;14
10	HD	1p32.3–1p32.3	50402893–50989867	586974	11;14
15	**BD**	1p32.3–1p32.3	50599579–51106763	507184	11;14
21	HD	1p32.2–1p31.1	50276000–73879000	23603000	4;14
22	**BD**	1p32.3–1p32.3	50924750–50971658	46908	4;14
23	HD	1p34.2–1p12	42342000–121700000	79358000	4;14
24	HD FAF1 only	1p33–1p32.3	49951000–50606000	655000	14;16
33	HD	1p36.33–1p12	1–119982000	119981999	6;14
34	HD	1p32.3–1p12	50750000–117611000	66861000	Hyper + IGH sep
35	HD	1p33–1p12	49064000–119989000	70925000	Hyper + IGH sep
40	HD	1p33–1p13.3	49723000–109237000	59514000	Hyper + IGH sep
51	HD	1p33–1p31.3	50018770–65125485	15106715	Hyper
52	**BD**	1p32.3–1p32.3	50925212–51007221	82009	Hyper
58	HD	1p32.3–1p13.3	50467681–107513627	57045946	Hyper
60	HD	1p34.1–1p32.2	45515071–55049538	9534467	Hyper
66	HD	1p36.33–1p12	1–119990000	119989999	Tetraploid
67	HD	1p36.33–1p12	1–119991000	119990999	Monosomy 13/14

Case	MAP3K14	Location	Breakpoints	Size (bp)	Primary
2	HD	17q21.31–17q21.32	44943000–47142000	2199000	11;14
36	HD	17q21.31–17q21.31	44583000–45982000	1399000	Hyper + IGH sep
44	HD	17p13.3–17q21.31	1–45734287	45734286	Hyper
45	Gain	17q21.31–17q21.31	45191000–45926000	735000	Hyper

Case	NFKB1 or NFKB2	Location	Breakpoints	Size (bp)	Primary
6	HD NFKB1	4q13.2–4q26	65930271–115942883	50012612	11;14
23	HD NFKB1	4p14–4q35.2	36402000–189875000	153473000	4;14
33	HD NFKB1	4p16.3–4q35.2	1–190214555	190214554	6;14
34	HD NFKB2	10q24.1–10q26.3	97005000–133797422	36792422	Hyper + IGH sep
40	HD NFKB1	4p16.3–4q35.2	1–190214555	190214554	Hyper + IGH sep
44	HD NFKB1	4q13.3–4q31.3	73221000–150520000	77299000	Hyper
46	HD NFKB2	10q24.32–10q24.33	101899000–103362000	1463000	Hyper
47	HD NFKB2	10q24.32–10q24.32	102148932–102721927	572995	Hyper
51	HD NFKB1	4p16.3–4q35.2	1–190214555	190214554	Hyper
65	HD NFKB2	10q24.32–10q25.1	102399071–104266176	1867105	Hyper
66	HD NFKB1	4p16.3–4q35.2	1–190214555	190214554	Tetraploid
66	HD NFKB2	10p15.3–10q26.3	1–133797422	133797421	Tetraploid
67	HD NFKB2	10q11.21–10q26.3	42354000–133797422	91443422	Monosomy 13/14

Case	TRAF2 or TRAF3	Location	Breakpoints	Size (bp)	Primary
8	HD TRAF3	14q22.3–14q32.33	56254000–104990000	48736000	11;14
23	HD TRAF3	14q11.2–14q32.33	19958000–105864169	85906169	4;14
32	**BD TRAF3**	14q32.32–14q32.32	102754161–102809688	55527	6;14
37	HD TRAF3	14q24.3–14q32.33	77344000–105590563	28246563	Hyper + IGH sep
44	HD TRAF3	14q21.1–14q32.33	39707000–107043718	67336718	Hyper
47	HD TRAF3	14q32.32–14q32.32	102845410–102902550	57140	Hyper
52	**BD TRAF3**	14q32.32–14q32.32	102722216–102790013	67797	Hyper
60	**BD TRAF3**	14q32.32–14q32.32	102741220–102888391	147171	Hyper
62	**BD TRAF3**	14q32.31–14q32.32	102680377–102913558	233181	Hyper
67	**BD TRAF3**	14q32.32–14q32.32	102841855–102878463	36608	Monosomy 13/14
67	HD TRAF2	9q34.3–9q34.3	136828276–136921241	92965	Monosomy 13/14

Case #	CRBN or IKZF1 or IKZF3	Location	Breakpoints	Size (bp)	Primary
8	HD IKZF3	17q12–17q21.31	35371000–44480000	9109000	11;14
17	HD CRBN	3p26.3–3p26.2	2738159–3194829	456670	4;14
22	HD CRBN	3p26.3–3q22.1	1–130531000	130530999	4;14
28	HD CRBN	3p26.3–3p25.2	1–12659000	12658999	14;20
41	HD CRBN	3p26.3–3p24.1	1–28213000	28212999	Hyper + IGH sep
44	HD IKZF3	17p13.3–17q21.31	1–45734287	45734286	Hyper
58	HD CRBN	3p26.3–3p26.1	1–7110000	7109999	Hyper
58	IKZF1 Gain + insertion to 10q25.2	17p12.2	50207542–50430511	222969	Hyper

Abnormalities of genes of known clinical significance in MM

Large gains of chromosome material are not indicated

*HD* heterozygous deletion, *BD* biallelic deletion indicated in bold, cytogenetic band and location in GRCh38

Evaluation for loss of function alterations of genes that have been associated with lenalidomide response or resistance (*CRBN*, *IKZF1* and *IKZF3*) identified 10.0% of the cohort had either a *CRBN*, *IKZF1* and *IKZF3* gene alteration (Table 5, Fig. 3, Supplemental Fig. 2D, case #58). Specifically, five cases had a heterozygous deletion of *CRBN* ranging in size from 457 Kb to 130.5 Mb including case #58 that had both a 7.1 Mb deletion encompassing *CRBN* and a 223 Kb *IKZF1* duplication with insertion of *IKZF1* into 10q25.2. Two cases had a heterozygous deletion that included *IKZF3* (9.1 Mb and 45.7 Mb).

## Discussion

Most clinical laboratories employ FISH analysis of CD138 enriched plasma cells as the preferred methodology in order to identify recurrent primary and secondary genomic abnormalities of prognostic and therapeutic significance in patients with PCNs^15^. The majority of these laboratories utilize only a limited FISH panel with many focusing on high risk abnormalities defined by the revised International Staging System (R-ISS) including 1q gain, t(4;14), t(14;16) or 17p deletion^15^. Some laboratories have incorporated the use of chromosomal microarray analysis in the detection of CNAs such as hyperdiploidy, 17p deletions and 1q gains, however microarray studies are unable to identify balanced structural rearrangements necessitating the use of other methodologies in the detection of IGH rearrangements^15^. It has also become increasingly apparent that some FISH probes, such as those targeting *MYC* rearrangements, display evidence of false negative results^18–22^. In addition, FISH panels for PCNs are variable between individual laboratories, provide a limited view of the whole genome and may not always reflect genomic complexity. Given that multiple research studies and investigational trials have used NGS based techniques to identify CNAs, SNVs along with structural rearrangements, we sought to explore the feasibility of employing an NGS technique in the detection of CNAs and structural rearrangements as a FISH replacement assay within a clinical genomics laboratory.

We describe the performance and added utility of a whole genome NGS based strategy, MPseq, in comparison to the current gold standard FISH approach in the evaluation of patients with PCNs. While MPseq and FISH displayed equal performance in the ability to classify the presence or absence of a recurrent, primary cytogenetic subtype (i.e. hyperdiploidy or specific IGH rearrangement), MPseq was superior compared to FISH in the characterization of rearrangement complexity, identification of secondary abnormalities, resolution of atypical FISH results and identification of novel abnormalities of prognostic significance not targeted by traditional FISH panels. Many samples chosen for this study had a high plasma cell burden (median 36% PCs) and ~33% of cases were obtained from fresh or frozen samples that did not require enrichment.

An advantage to using a whole genome NGS technique like MPseq is the ability to identify rearrangements using an unbiased approach. Other laboratories have developed and validated NGS methodologies utilizing target-enrichment approaches for PCNs allowing a custom target pull down of limited genomic regions^13,35–37^. While these targeted approaches have reduced cost and simplified analysis workflows, a genome wide approach utilizing long-insert whole genome sequencing employed by the MMRF CoMMpass Study in their Seq-FISH analysis has demonstrated improved sensitivity with similar specificity in relation to clinical FISH testing^38^. Although MPseq is similar to Seq-FISH with regard to a whole genome sequencing approach, a significant limitation to the current MPseq strategy is the inability to identify SNVs. This limitation can be resolved with deeper and faster sequencing, coupled with reduced sequencing costs. An integrated genomic analysis incorporating structural variation, CNAs, and SNVs together may lead to enhanced prognostication^13^. Of practical consideration is the ~two-fold increased cost and “turn-around-time” of reporting of clinical grade testing for MPseq compared to a comprehensive FISH panel; although we anticipate over time the cost and time of reporting for NGS approaches will continue to be reduced.

Another limitation to the use of MPseq is the inability to identify rearrangements in highly repetitive regions of the genome containing constitutive heterochromatin such as those involving telomeres, centromeres, and in regions near the centromeres of chromosomes 1, 9, and 16 and in the Y chromosome^27^. This limitation may be reflected by the inability of MPseq to identify apparent trisomies in 2 cases (cases 5 and 42) with evidence of hyperdiploidy. Case 5 displayed a gain of a structurally abnormal chromosome 3 by conventional chromosome studies. Since the centromere regions that are targeted by the FISH probes are not covered by MPseq, it is unclear whether a small gain or presence of a polymorphism of these regions are present without evidence of a bona fide trisomy or whether the trisomy was present at a subclonal level below the limit of detection by MPseq (<25% for CNAs)^18^. Polymorphisms of the acrocentric chromosome 15 have also been reported^39^ and are observed in FISH analysis of PCNs in our laboratory (data not shown). Discrepancies involving chromosome 15 are present in 6 of 70 cases in this study demonstrated by either a monosomy 15 FISH result with normal chromosome 15 s by MPseq or either a normal or monosomy 15 FISH result with trisomy 15 by MPseq. Since MPseq does not rely on detection of only the centromere region like FISH, analysis of copy number changes throughout the whole chromosome can be useful to interpret the presence or absence of a trisomy. On the other hand, any missed hyperdiploid cases may be of less relevance as hyperdiploidy can be detected by flow cytometric methods^40^.

MPseq is not currently used in the detection of copy-neutral LOH or ploidy. As a result, MPseq did not identify any cases as tetraploid, an abnormality found in approximately 6% of patients with MM that has been associated with high-risk genomic abnormalities and with poor prognosis in NDMM^41^. Six patients in our cohort had evidence of tetraploidy and 5 of these cases had tetraploidy in combination with high risk cytogenetics (Fig. 1), which were identified by MPseq. For case 39, it is unclear why MPseq failed to identify a 1q gain. No evidence of a duplication involving chromosome 1q (chr. 1:155122503–155571708) was identified by MPseq and based on FISH data, the 1q gain did not appear to be subclonal, however this sample was extracted from unsorted bone marrow with 20% clonal plasma cells which may have contributed to this missed abnormality. Subclonal CNAs and cases with low tumor load have a risk of being missed by MPseq, a risk that also exists when performing FISH.

Of the 30 discordant cases, 19 cases had abnormalities identified by MPseq that were missed by FISH, the majority involving insertional events near the *MYC* gene region. In total, *MYC* rearrangements were found in 51.4% of the cases in our cohort which includes NDMM and RRMM. This is consistent with previous reports identifying *MYC* rearrangements in 35% of NDMM with increased frequency in RRMM^10,42^. *MYC* rearrangements have been reported as subclonal events and are associated with disease progression^3,11,43^. Approximately 66% of *MYC* rearrangements have been found in association with non-Ig partners resulting in juxtaposition to enhancer sequences promoting aberrant *MYC* gene expression, which may be targeted by BRD4 inhibitors in MM^10^. Identification of *MYC* rearrangements using a break-apart probe strategy resulted in a 50.0% false negative rate in our patient cohort. Whether these false negative insertion cases have the same prognostic implication as other *MYC* rearrangements remains unknown.

Two cases had deletions of the *TP53* gene region that were not identified by FISH. For cases 4 and 21, MPseq identified a deletion of *TP53* (5.6 Mb in case 4, 2.7 Mb in case 21). Interestingly, for case 21, MPseq also identified a translocation involving *TP53* (to 4q32.1). Both cases were scored as having two copies of *TP53* by FISH and represent false negative results due the location of the deletion in relation to the FISH footprint in case 4 and the *TP53* translocation in combination with the deletion in case 21. Although these cases had missed high risk abnormalities, the mSMART risk did not change since those cases also had additional high risk abnormalities [1q gain for cases 4 and t(4;14) for case 21]. For case 4, a separate NGS assay analyzing SNVs identified a pathogenic *TP53* mutation [Chr17(GRCh37):g.7577111 G > T; NM_001126113.2(TP53):c.827 C > A; p.Ala276Asp] located in the DNA-binding domain and in vitro functional data predicts that this variant results in non-functional p53 protein^44^. *TP53* has been found to be mutated in 3–16% of NDMM^6,45–47^ with a higher frequency in RRMM^48–50^. *TP53* mutations in combination with 17p deletions are associated with double hit MM with reduced overall, progression-free and relapse-free survival^51^. Therefore, this missed *TP53* deletion fails to identify the presence of a likely double hit MM in patient 4. The combination of a rearrangement and deletion also likely represents a double hit MM abnormality in case 21.

The *CCND1/IGH* dual color, dual fusion probe set is used to identify *CCND1/IGH* rearrangements. However, three copies of *CCND1* in the absence of IGH fusion can indicate trisomy 11 or non-IGH *CCND1* rearrangements. MPseq identified three *CCND1* rearrangements including case 4 (*IGL/CCND1*), case 43 (*IGK/CCND1*) described more fully in Peterson, et al.^52^ and case 57 (*BRINP3/CCND1*). FISH also identified amplification of *CCND1* in case 4, three copies of *CCND1* in case 43 and a normal signal pattern for *CCND1* in case 57. The *CCND1* rearrangement identified in case 57 was a complex translocation between 1q31.1 and 11q13.3 consisting of four junctions and deletions of ~100 kb at both ends. Through this complex event, *CCND1* is brought into close proximity to the 3′ end of *BRINP3*, while the balancing set of junctions brings the 5′ end of *BRINP3* near 11q24.3. Additionally, the derivative chromosome containing *CCND1* has been copied. Overall this would result in three copies of *CCND1*, two of which have been translocated near the 3′ end of *BRINP3*. This case demonstrates how MPseq is able to determine complex rearrangements involving important genes without prior knowledge of the junction partner location.

Although immunoglobulin lambda rearrangements have been recently reported in association with poor prognosis^3^, light chain rearrangements are typically not evaluated in the diagnostic work up of MM in most clinical genomics laboratories. Using MPseq data, we identify 10 cases (14.3% of entire cohort) with IGL rearrangements with five of these cases with standard risk cytogenetic results. IGL rearrangements and other focal deletions of clinical significance are typically not evaluated by FISH. Given the high rate of false-negative *MYC* rearrangements and inability to appreciate all abnormalities of clinical significance, we demonstrate that MPseq has increased clinical value compared to FISH in characterizing genomic abnormalities in PCNs.

## Supplementary information

Supplemental data

Supplemental Figures

## References

[1] Howlader N (2010). Improved estimates of cancer-specific survival rates from population-based data. J. Natl Cancer Inst..

[2] Rajkumar SV (2018). Multiple myeloma: 2018 update on diagnosis, risk-stratification, and management. Am. J. Hematol..

[3] Barwick BG (2019). Multiple myeloma immunoglobulin lambda translocations portend poor prognosis. Nat. Commun..

[4] Chapman MA (2011). Initial genome sequencing and analysis of multiple myeloma. Nature..

[5] Egan JB (2012). Whole-genome sequencing of multiple myeloma from diagnosis to plasma cell leukemia reveals genomic initiating events, evolution, and clonal tides. Blood..

[6] Lohr JG (2014). Widespread genetic heterogeneity in multiple myeloma: implications for targeted therapy. Cancer Cell..

[7] Miller A (2017). High somatic mutation and neoantigen burden are correlated with decreased progression-free survival in multiple myeloma. Blood Cancer J..

[8] Walker BA (2018). Identification of novel mutational drivers reveals oncogene dependencies in multiple myeloma. Blood..

[9] Walker BA (2015). APOBEC family mutational signatures are associated with poor prognosis translocations in multiple myeloma. Nat. Commun..

[10] Affer M (2014). Promiscuous MYC locus rearrangements hijack enhancers but mostly super-enhancers to dysregulate MYC expression in multiple myeloma. Leukemia..

[11] Misund K. K., et al. MYC dysregulation in the progression of multiple myeloma. *Leukemia* (2019). https://www.ncbi.nlm.nih.gov/pubmed/31439946. [Epub ahead of print] 10.1038/s41375-019-0543-4 PMC6923575 31439946

[12] Manier S (2017). Genomic complexity of multiple myeloma and its clinical implications. Nat. Rev. Clin. Oncol..

[13] Bolli N (2018). Analysis of the genomic landscape of multiple myeloma highlights novel prognostic markers and disease subgroups. Leukemia..

[14] Rajkumar S. V. mSMART stratification for myeloma and risk-adapted therapy. www.msmart.org

[15] Pugh TJ (2018). Assessing genome-wide copy number aberrations and copy-neutral loss-of-heterozygosity as best practice: an evidence-based review from the Cancer Genomics Consortium working group for plasma cell disorders. Cancer Genet-Ny.

[16] Kumar SK, Rajkumar SV (2018). The multiple myelomas - current concepts in cytogenetic classification and therapy. Nat. Rev. Clin. Oncol..

[17] Sonneveld P (2016). Treatment of multiple myeloma with high-risk cytogenetics: a consensus of the International Myeloma Working Group. Blood..

[18] Aypar U (2019). Mate pair sequencing improves detection of genomic abnormalities in acute myeloid leukemia. Eur. J. Haematol..

[19] King Rebecca L., McPhail Ellen D., Meyer Reid G., Vasmatzis George, Pearce Kathryn, Smadbeck James B., Ketterling Rhett P., Smoley Stephanie A., Greipp Patricia T., Hoppman Nicole L., Peterson Jess F., Baughn Linda B. (2018). False-negative rates for MYC fluorescence in situ hybridization probes in B-cell neoplasms. Haematologica.

[20] Peterson JF (2019). Characterization of a cryptic IGH/CCND1 rearrangement in a case of mantle cell lymphoma with negative CCND1 FISH studies. Blood Adv..

[21] Peterson Jess F., Pitel Beth A., Smoley Stephanie A., Smadbeck James B., Johnson Sarah H., Vasmatzis George, Kearney Hutton M., Greipp Patricia T., Hoppman Nicole L., Baughn Linda B., Ketterling Rhett P. (2019). Use of mate-pair sequencing to characterize a complex cryptic BCR/ABL1 rearrangement observed in a newly diagnosed case of chronic myeloid leukemia. Human Pathology.

[22] Peterson Jess F., Pitel Beth A., Smoley Stephanie A., Smadbeck James B., Johnson Sarah H., Vasmatzis George, Koon Sarah J., Webley Matthew R., McGrath Mary, Bayerl Michael G., Baughn Linda B., Rowsey Ross A., Ketterling Rhett P., Greipp Patricia T., Hoppman Nicole L. (2019). Detection of a cryptic NUP214/ABL1 gene fusion by mate-pair sequencing (MPseq) in a newly diagnosed case of pediatric T-lymphoblastic leukemia. Molecular Case Studies.

[23] Walker BA (2014). Translocations at 8q24 juxtapose MYC with genes that harbor superenhancers resulting in overexpression and poor prognosis in myeloma patients. Blood. Cancer J..

[24] Walker BA (2013). Characterization of IGH locus breakpoints in multiple myeloma indicates a subset of translocations appear to occur in pregerminal center B cells. Blood..

[25] Baughn LB (2018). Differences in genomic abnormalities among African individuals with monoclonal gammopathies using calculated ancestry. Blood Cancer J..

[26] Jang JS (2019). Molecular signatures of multiple myeloma progression through single cell RNA-Seq. Blood Cancer J..

[27] Johnson SH (2018). SVAtools for junction detection of genome-wide chromosomal rearrangements by mate-pair sequencing (MPseq). Cancer Genet.

[28] Smadbeck James B., Johnson Sarah H., Smoley Stephanie A., Gaitatzes Athanasios, Drucker Travis M., Zenka Roman M., Kosari Farhad, Murphy Stephen J., Hoppman Nicole, Aypar Umut, Sukov William R., Jenkins Robert B., Kearney Hutton M., Feldman Andrew L., Vasmatzis George (2018). Copy number variant analysis using genome-wide mate-pair sequencing. Genes, Chromosomes and Cancer.

[29] Drucker TM (2014). BIMA V3: an aligner customized for mate pair library sequencing. Bioinformatics..

[30] Gaitatzes A, Johnson SH, Smadbeck JB, Vasmatzis G (2018). Genome U-Plot: a whole genome visualization. Bioinformatics..

[31] Lee N (2017). Discrepancies between the percentage of plasma cells in bone marrow aspiration and BM biopsy: Impact on the revised IMWG diagnostic criteria of multiple myeloma. Blood Cancer J..

[32] Demchenko Y (2016). Frequent occurrence of large duplications at reciprocal genomic rearrangement breakpoints in multiple myeloma and other tumors. Nucleic Acids Res.

[33] Keats JJ (2007). Promiscuous mutations activate the noncanonical NF-kappaB pathway in multiple myeloma. Cancer Cell..

[34] Zhu YX (2017). Loss of FAM46C promotes cell survival in myeloma. Cancer Res..

[35] Jimenez C (2017). A next-generation sequencing strategy for evaluating the most common genetic abnormalities in multiple myeloma. J. Mol. Diagn..

[36] Bolli N (2016). A DNA target-enrichment approach to detect mutations, copy number changes and immunoglobulin translocations in multiple myeloma. Blood Cancer J..

[37] White BS (2018). A multiple myeloma-specific capture sequencing platform discovers novel translocations and frequent, risk-associated point mutations in IGLL5. Blood Cancer J..

[38] Goldsmith Scott R., Fiala Mark A., Dukeman James, Ghobadi Armin, Stockerl-Goldstein Keith, Schroeder Mark A., Tomasson Michael, Wildes Tanya M., Vij Ravi (2019). Next Generation Sequencing-based Validation of the Revised International Staging System for Multiple Myeloma: An Analysis of the MMRF CoMMpass Study. Clinical Lymphoma Myeloma and Leukemia.

[39] Davila-Rodriguez MI (2011). Constitutive heterochromatin polymorphisms in human chromosomes identified by whole comparative genomic hybridization. Eur. J. Histochem..

[40] Sidana S (2019). Rapid assessment of hyperdiploidy in plasma cell disorders using a novel multi-parametric flow cytometry method. Am. J. Hematol..

[41] Sidana S (2019). Tetraploidy is associated with poor prognosis at diagnosis in multiple myeloma. Am. J. Hematol..

[42] Shou Y (2000). Diverse karyotypic abnormalities of the c-myc locus associated with c-myc dysregulation and tumor progression in multiple myeloma. Proc. Natl Acad. Sci. USA.

[43] Glitza IC (2015). Chromosome 8q24.1/c-MYC abnormality: a marker for high-risk myeloma. Leuk. Lymphoma..

[44] Kato S (2003). Understanding the function-structure and function-mutation relationships of p53 tumor suppressor protein by high-resolution missense mutation analysis. Proc. Natl Acad. Sci USA.

[45] Bolli N (2014). Heterogeneity of genomic evolution and mutational profiles in multiple myeloma. Nat. Commun..

[46] Kortum KM (2015). Longitudinal analysis of 25 sequential sample-pairs using a custom multiple myeloma mutation sequencing panel (M(3)P). Ann. Hematol..

[47] Walker BA (2015). Mutational spectrum, copy number changes, and outcome: results of a sequencing study of patients with newly diagnosed myeloma. J. Clin. Oncol..

[48] Weinhold N (2016). Clonal selection and double-hit events involving tumor suppressor genes underlie relapse in myeloma. Blood..

[49] Xiong W (2008). An analysis of the clinical and biologic significance of TP53 loss and the identification of potential novel transcriptional targets of TP53 in multiple myeloma. Blood..

[50] Chng WJ (2007). Clinical significance of TP53 mutation in myeloma. Leukemia..

[51] Walker BA (2019). A high-risk, Double-Hit, group of newly diagnosed myeloma identified by genomic analysis. Leukemia..

[52] Peterson JF (2019). Whole genome mate-pair sequencing of plasma cell neoplasm as a novel diagnostic strategy: a case of unrecognized t (2; 11) structural variation. Clin. Lymphoma, Myeloma Leuk..

