# Supplementary material for: Mate pair sequencing outperforms fluorescence in situ hybridization in the genomic characterization of multiple myeloma
Source: Blood Cancer J. 2019 Dec 16;9(12):103. doi: 10.1038/s41408-019-0255-z (PMC6914798; doi:10.1038/s41408-019-0255-z)
Supplement: Supplementary file 1 — Supplemental data [file 41408_2019_255_MOESM1_ESM.docx]

**Mate pair sequencing outperforms fluorescence in situ hybridization in the genomic characterization of multiple myeloma**

James Smadbeck, Ph.D.^1*^, Jess F. Peterson, M.D.^2*^, Kathryn E. Pearce, M.S. ^2^, Beth A. Pitel, M.S.^2^, Andrea Lebron Figueroa^2^, Michael Timm, B.S. ^3^, A. Dragan Jevremovic, M.D. ^3^, Ph.D., Min Shi, M.D. ^3^, Keith Stewart, M.B., Ch.B. ^4^, Esteban Braggio, Ph.D. ^4^, Daniel L. Riggs, Ph.D. ^4^, P. Leif Bergsagel, M.D. ^4^, George Vasmatzis, Ph.D. ^1^, Hutton M. Kearney, Ph.D.^2^, Nicole L. Hoppman, Ph.D.^2^, Rhett P. Ketterling, M.D.^2^, Shaji Kumar, M.D.^5^, S. Vincent Rajkumar, M.D.^5^, Patricia T. Greipp, D.O. ^2^, Linda B. Baughn, Ph.D.^2^

*****contributed equally to this work

**Running Title:** Detection of genomic abnormalities in MM by NGS

^1^Center for Individualized Medicine-Biomarker Discovery, Mayo Clinic, Rochester, MN;

^2^Division of Laboratory Genetics, Department of Laboratory Medicine and Pathology, Mayo Clinic Rochester, MN;

^3^Division of Hematopathology, Department of Laboratory Medicine and Pathology, Mayo Clinic, Rochester, MN;

^4^Division of Hematology, Department of Internal Medicine, Mayo Clinic, Scottsdale, AZ;

^5^Division of Hematology, Department of Internal Medicine, Mayo Clinic, Rochester, MN

**Supplemental Information Text Summary: Data includes supplemental methods, two supplemental figures with figure legends, and three supplemental tables.**

**Supplemental Methods:**

**Conventional chromosome analysis**

BM cells were cultured, harvested and banded utilizing standard cytogenetic techniques according to specimen-specific protocols. When available, 20 metaphases were analyzed and results reported per 2016 International System for Human Cytogenomic Nomenclature (ISCN).

**Flow Cytometry**

Abnormal PC percentage was obtained using flow cytometry. BM specimens were spun down and the pellet is lysed using 14 mL of ACK lysing buffer (Thermo Fisher Scientific), followed by two washes with PBS. The cell pellet is then re-suspended in 3% BSA/PBS with Azide (BD Pharmingen) and stained with the following antibodies: CD138 PerCPcy5.5, CD19 PE-cy7, CD38 FITC, CD45 APC-H7 (all BD Biosciences) for 15 minutes. Following the wash in Caltag A reagent (Thermo Fisher Scientific) for 10 minutes, the pellet is re-suspended in Caltag B reagent for permeabilization. Antibodies for cytoplasmic staining are added (Kappa APC and Lambda PE – both from Dako North America Inc.), and the specimen is incubated for 20 minutes. This is followed by the wash step and the cell pellet is re-suspended in 500 μL of PBS. The flow cytometry FCS files are obtained on BD FACSCanto™ II instruments (100,000 events per specimen). The files are analyzed using Kaluza software (Beckman Coulter).

**Supplemental Figure Legends:**

**Supplemental Figure 1: Schematic of MM workflow.** Bone marrow samples are processed for FISH analysis and analyzed for MPseq, DNA is extracted from fixed cell pellets (FCP), fresh or frozen bone marrow or from cells subjected to magnetic enrichment using antibodies to CD138 or flow cytometry. Genomic DNA is prepared using the Illumina Nextera Mate Pair library preparation kit and sequenced on the HiSeq 2500. Reads are aligned to the reference genome with BIMA and variants detected using SVAtools. Variant detection is achieved using junction detection and CNVDetect algorithms.

**Supplemental Figure 2: Examples of observed secondary abnormalities.** A) focal biallelic deletion of *FAF1/CDKN2C* via overlapping deletions on 1p. B) focal biallelic deletion of *TRAF3* via overlapping deletions on 14q. C) focal biallelic deletion of *CYLD* via overlapping deletions on 16q. D) heterozygous deletion of *CRBN* via terminal deletion of 3p. In all cases junctions detected by MPseq are shown as magenta lines. Regions of normal 2N copy number level are not colored and copy number level is depicted by a gray line, regions of single deletion are shaded light red and copy number level is depicted as a red line, and regions of homozygous deletion are shaded darker red and copy number level is depicted as a red line. Case # is given at the top, y-axis gives the chromosome number, and x-axis gives the genomic position in Mb (GRCh38).

Supplemental table 1: PCN FISH panel

| **Recurrent primary abnormalities** |
| --- |
| *IGH rearrangement* |
| t(11;14), *CCND1*/*IGH* |
| t(4;14)(p16.3;q32) *FGFR3*/*IGH* |
| t(14;16)(q32;q23) *IGH*/*MAF* |
| t(14;20)(q32;q12) *IGH*/*MAFB* |
| t(6;14)(p21;q32) *CCND3*/*IGH* |
| Hyperdiploidy (gains of chr. 3,7,9,11,15,17) |
| **Recurrent secondary abnormalities** |
| Monosomy 17/17p deletion |
| Monosomy 13/13q deletion |
| 1q gain |
| *MYC* rearrangement |

| **Panel** | **Probe** | **Type** | **Source** | **Footprints** | **Footprints** | **Footprints** | **Footprints** | **Footprints** |
| --- | --- | --- | --- | --- | --- | --- | --- | --- |
| 1 | IGH | Break apart | In house | chr14:105015261-105657128 (3' IGH) | chr14:105727280-105898654 (3' IGH) | chr14:106210572-106340055 (5' IGH) | chr14:106375021-106860217 (5' IGH) |  |
| 1 | t(11;14) | Dual fusion | Abbott Molecular | chr14:105199125-106860200 (IGH) | chr11:68839085-69781386 (CCND1) |  |  |  |
| 1 | *TP53*/17 CEN | Enumeration | Abbott Molecular | chr17:7591077-7762219 (TP53) | CEN 17 |  |  |  |
| 1 | *RB1/LAMP1* 13q | Enumeration | Abbott Molecular | chr13:48161337-48583237 (RB1) | chr13:113037008-113649120 (LAMP1) |  |  |  |
| 1 | CEN 9/CEN 15 | Enumeration | Abbott Molecular | CEN 9 | CEN 15 |  |  |  |
| 1 | CEN 3/CEN 7 | Enumeration | Abbott Molecular | CEN 3 | CEN 7 |  |  |  |
| 1 | *MYC* | Break apart | Abbott Molecular | chr8:127351112-127628392 (5'MYC) | chr8:129257504-129664187 (3'MYC) |  |  |  |
| 1 | 1p/1q | Enumeration | In house | chr1:3623190-3624743 (TP73) | chr1:3648554-3800166 (TP73) | chr1:155122503-155571708 (1q22) |  |  |
| 2 | t(14;16) | Dual fusion | Abbott Molecular | chr14:105199125-106860200 (IGH) | chr16:77138613-77488295 (MAF) | chr16:79698612-80044450 (MAF) |  |  |
| 2 | t(4;14) | Dual fusion | Abbott Molecular | chr14:105199125-106860200 (IGH) | chr4:1316818-2349963 (FGFR3) |  |  |  |
| 2 | t(14;20) | Dual fusion | Abbott Molecular | chr14:105199125-106860200 (IGH) | chr20:39281050-39771307 (MAFB) | chr20:39771598-39916467 (MAFB) | chr20:39919268-40223267 (MAFB) | chr20:40251152-40605425 (MAFB) |
| 2 | t(6;14) | Dual fusion | Abbott Molecular | chr14:105199125-106860200 (IGH) | chr6:41704407-42096473 (CCND3) |  |  |  |
|  |  |  |  |  |  |  |  |  |
|  | Location of FISH probes in GRCh38 | |  |  |  |  |  |  |

Supplemental table 2: FISH probes and genomic footprints

Supplemental table 3: Conventional chromosome results

| **Case #** | **Primary abnormality (FISH)** | **Karyotype** |
| --- | --- | --- |
| 1 | 11;14 | 39-40,XY,del(6)(q13q25),-8,t(11;14)(q13;q32),-12,-13,-16,-22[cp3]/46,XY[8] |
| 2 |  | ND |
| 3 |  | ND |
| 4 |  | 46,XY[20] |
| 5 |  | 48,XY,+add(1)(p22),+add(3)(p21),-6,i(8)(q10),+9,t(11;14)(q13;q32),-13,+15,+19[cp2]/46,XY[18] |
| 6 |  | ND |
| 7 |  | ND |
| 8 |  | 44-46,XY,-9,-11,der(14)t(11;14)(q13;q32)x1-2,-16,-17,+2-4mar[cp4]/46,XY[16] |
| 9 |  | 92,XXYY,t(1;14)(p32;q32),t(11;14)(q13;q32)x2,del(16)(q11.2)x2[3]/46,XY[17] |
| 10 |  | 46,XY[20] |
| 11 |  | 46,XY[20] |
| 12 |  | ND |
| 13 |  | 46,XX [20] |
| 14 |  | 46,XX[20] |
| 15 |  | 40-43,X,-X,add(1)(p13)[2],der(5)t(1;5)(q21;q31),-11,-14,der(14)t(11;14)(q13;q32),  -16,-17,+2mar[cp3]/46,XX[17] |
| 16 | 4;14 | 46,XX[20] |
| 17 |  | ND |
| 18 |  | 41,X,dic(Y;1)(q12;p13),+1,t(2;20)(p21;q13.1),-6,-8,-12,-13,-22[1]/46,XY[19] |
| 19 |  | 46,XY[20] |
| 20 |  | 44,XY,add(2)(p13),add(3)(p21),add(5)(q15),add(6)(q13),add(8)(p11.2),-13,der(16)t(1;16)(q11;q22),add(17)(p11.2),-20,-22,+mar[1]/46,XY[19] |
| 21 |  | ND |
| 22 |  | ND |
| 23 |  | ND |
| 24 | 14;16 | ND |
| 25 |  | ND |
| 26 |  | 47,XX,+der(1;5)(q10;p10)[4]/46,XX[16] |
| 27 |  | 46,XX [20] |
| 28 | 14;20 | 79-80,XX,-X,-X,-1,dic(1;2)(p13;q31)x2,der(1)t(1;1)(p11;q42),add(2)(q31),der(3;22)(q10;q10)x2,der(6)t(6;13)(q21;q12)x2,-8,-8,add(9)(p13)x2,add(10)(p11.2)x2,-12,-13,-13,-13,-13,t(14;20)(q32;q12)x2,hsr(14)(p11.2)x2,-16,-16,+1-5mar[cp15]/46,XX[5] |
| 29 |  | 42,X,-Y,del(1)(p13p32),add(7)(q32),add(8)(p11.2),add(9)(p13),-13,t(14;20)(q32;q12),-16,-20[11]/45,X,-Y[9] |
| 30 |  | 40-43,-X,add(X)(q22),t(1;8)(p13;q24.3),del(3)(q25),-13,t(14;20)(q32;q12),-del(16)(q12),18,-21,-22[cp20] |
| 31 |  | ND |
| 32 | 6;14 | 44,XY,+add(1)(q21),del(1)(p12p32)x2,-6,-13,der(14)t(6;14)(p21;q32),add(17)(p11.2),add(19)(q13.3)[6]/46,XY[12] |
| 33 |  | 84,XX,der(Y;16)(q10;p10)x2,i(1)(q10)x2,-2,-4,-4,t(6;14)(p21.1;q32)x2,-8,t(8;14)  (q24.1;q32),-10,-11,der(14)t(8;14)[6]/46,XY[14] |
| 34 | Hyperdiploidwith IGH separation | 45-49,XX,add(1)(p11)x1-2,+add(1)(q21),add(4)(q12),ins(5;?)(q13;?),der(6)t(6;14)(p21;q32)add(6)(q13),add(7)(q36)x1-2,+8,add(8)(q24.1)x1-2,add(10)(q22),add(10)(q22),add(11)(q25),-13,der(14)t(6;14),+15,der(15;15)(q10;q10),del(16)(q11.2),del(16)(q12),add(20)(q11.2),add(20)(q13.3)[cp19]/46,XX[1] |
| 35 |  | 51-52,X,-X,del(1)(p13p32),add(2)(q37),+add(3)(p13),add(4)(p12),+5,der(8;13)(q10;q10),add(8)(q24.1),+9,del(10)(q24),+idic(11)(p11.2),+15,-16,der(17)t(X;17)(q13;p11.2),+18,der(20)t(11;20)(q13;q11.2),+21,+1-3mar[cp13]/46,XX[7] |
| 36 |  | ND |
| 37 |  | ND |
| 38 |  | 46,XX [20] |
| 39 |  | 46,XY[20] |
| 40 |  | ND |
| 41 |  | 46,XY[20] |
| 42 |  | 46,XY[20] |
| 43 | Hyperdiploid | ND |
| 44 |  | ND |
| 45 |  | 46,XY[20] |
| 46 |  | ND |
| 47 |  | ND |
| 48 |  | 46,XX[20] |
| 49 |  | ND |
| 50 |  | ND |
| 51 |  | ND |
| 52 |  | ND |
| 53 |  | 46,XX [20] |
| 54 |  | ND |
| 55 |  | 46,XY[20] |
| 56 |  | ND |
| 57 |  | ND |
| 58 |  | ND |
| 59 |  | 46,XY[20] |
| 60 |  | 46,XY[10] |
| 61 |  | 46,XX [20] |
| 62 |  | 46,XY[20] |
| 63 |  | 46,XX [20] |
| 64 |  | 46,XX [20] |
| 65 |  | 46,XX [20] |
| 66 | Tetraploid | ND |
| 67 | Monosomy 13/14 | 45,X,-Y[7]/46,XY[13] |
| 68 | Monosomy 15 | 46,XY[20] |
| 69 |  | 46,XX [20] |
| 70 | Normal | 46,XX [20] |

Cytogenetic abnormalities identified by conventional chromosome studies. Total cells analyzed in brackets. ND: Not done.
