# Supplementary material for: Mate pair sequencing outperforms fluorescence in situ hybridization in the genomic characterization of multiple myeloma
Source: Blood Cancer J. 2019 Dec 16;9(12):103. doi: 10.1038/s41408-019-0255-z (PMC6914798; doi:10.1038/s41408-019-0255-z)
Supplement: Supplementary file 2 — Supplemental Figures [file 41408_2019_255_MOESM2_ESM.pptx]

## Slide 1
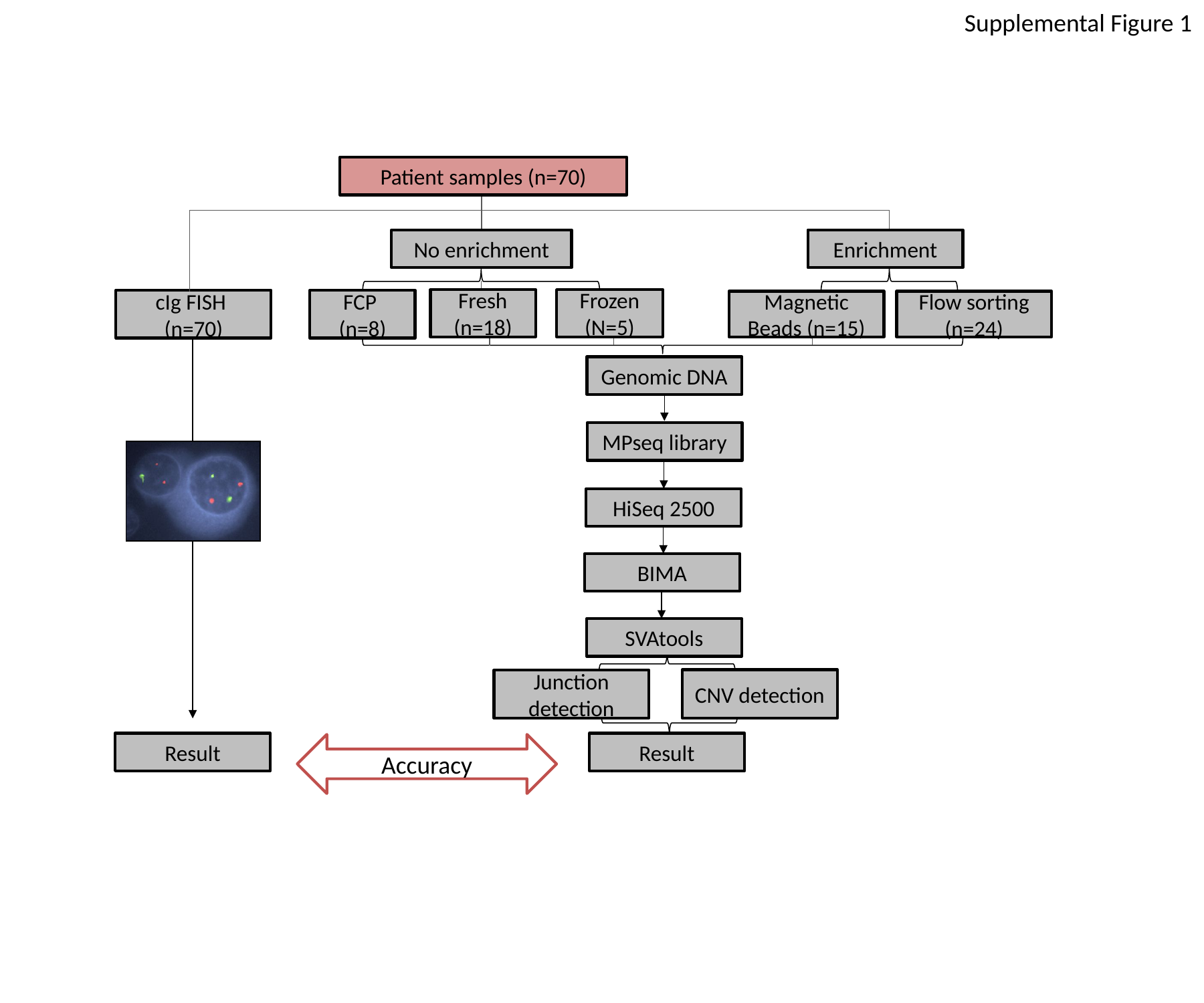

Supplemental Figure 1
Patient samples (n=70)
No enrichment
Enrichment
Fresh (n=18)
Frozen (N=5)
cIg FISH
(n=70)
FCP
(n=8)
Flow sorting (n=24)
Magnetic Beads (n=15)
Genomic DNA
MPseq library
HiSeq 2500
BIMA
SVAtools
CNV detection
Junction detection
Result
Result
Accuracy

## Slide 2
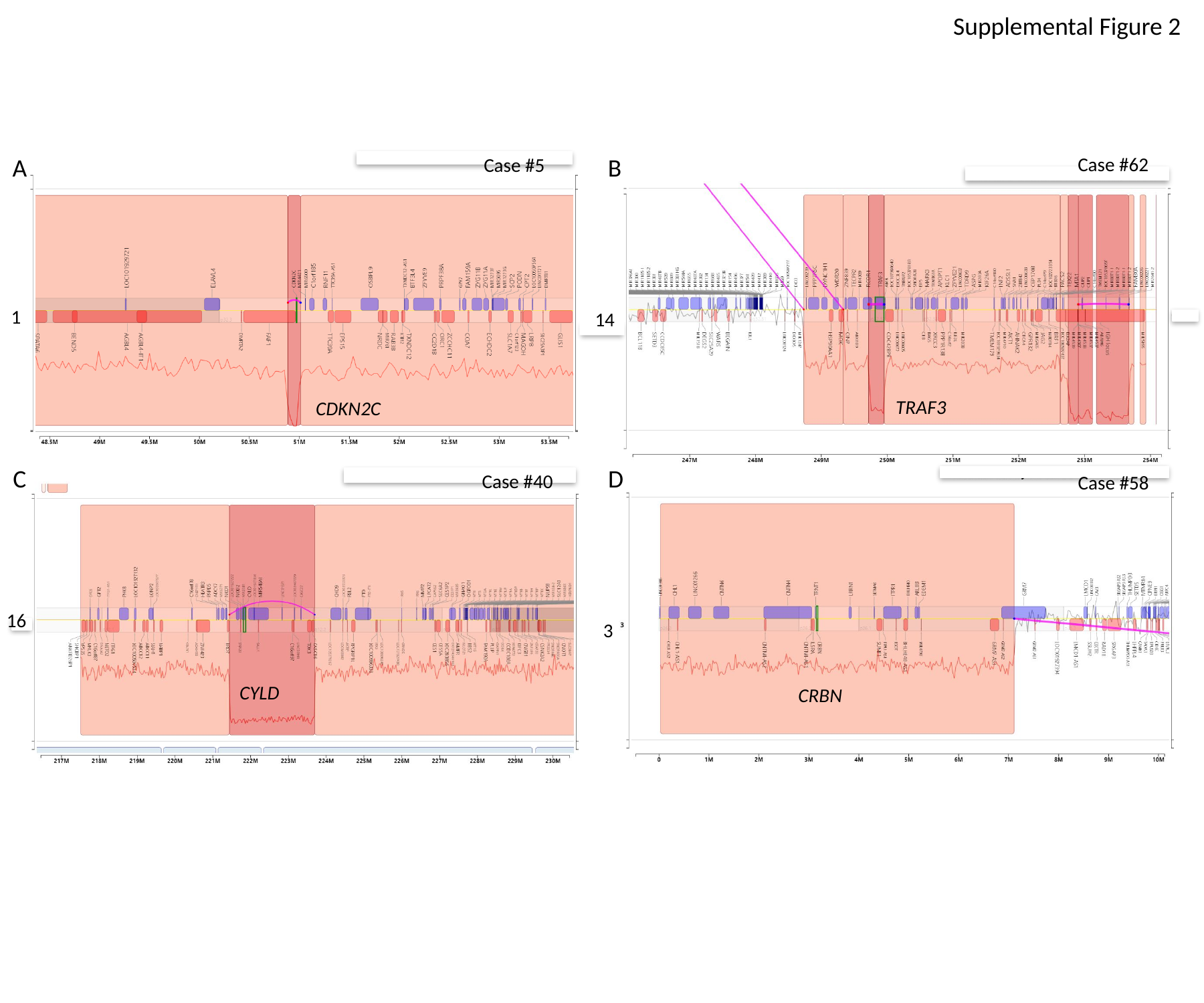

Supplemental Figure 2
Case #62
A
B
Case #5
1
14
TRAF3
CDKN2C
C
D
Case #40
Case #58
16
3
CYLD
CRBN
